# BIN1 and ALDH1B1 Deficiency in Colonic Smooth Muscle Drives Mitochondrial Dysfunction and Fibrosis in Slow‐Transit Constipation

**DOI:** 10.1002/advs.202523688

**Published:** 2026-06-12

**Authors:** Jianbo Liu, Hao Zhang, Wenhao Qiao, Ran Liu, Jie Sun, Xiaopei Li, Qin Li, Dongbo Zhao, Dawei Chen, Jingxin Li, Shuxiao Dong

**Affiliations:** ^1^ Department of Gastrointestinal Surgery, Shandong Provincial Third Hospital Shandong University Jinan Shandong China; ^2^ Department of Physiology, School of Basic Medical Sciences, Cheeloo College of Medicine Shandong University Jinan Shandong China; ^3^ Department of Gastroenterology, Shandong Provincial Third Hospital Shandong University Jinan Shandong China; ^4^ Department of Thoracic Surgery, Shandong Cancer Hospital and Institute Shandong First Medical University and Shandong Academy of Medical Sciences Jinan Shandong China; ^5^ Institute of Clinical Molecular Biology Kiel University Kiel Germany; ^6^ School of Basic Medicine and Clinical Pharmacy China Pharmaceutical University Nanjing China; ^7^ Institute of Translational Medicine China Pharmaceutical University Nanjing China; ^8^ Shandong Key Laboratory of Mental Disorders and Intelligent Control Jinan Shandong China

**Keywords:** fibrosis, intestinal motility, mitochondrial function

## Abstract

Slow‐transit constipation (STC) is a disabling motility disorder with unclear smooth‐muscle mechanisms. Through spatial proteomics of human colon and functional assays in primary human colonic smooth muscle cells (HCoSMCs), we identified Bridging Integrator 1 (BIN1) and Aldehyde Dehydrogenase 1B1 (ALDH1B1) as key regulators of intestinal motility. Both proteins were markedly reduced in smooth muscle from STC patients and localized to fibrotic areas. Lentiviral knockdown of BIN1 or ALDH1B1 impaired ATP‐evoked Ca^2^^+^ responses and contraction, disrupted mitochondrial architecture, and increased reactive oxygen species. BIN1 deficiency activated mitochondrial apoptosis and extracellular matrix deposition, whereas ALDH1B1 loss induced mitophagy and NF‐κB–driven inflammation. Transcriptomic and histological analyses confirmed convergence on profibrotic pathways. Together, these findings reveal a smooth‐muscle‐centric mechanism underlying STC pathogenesis and nominate BIN1 and ALDH1B1 as promising therapeutic entry points to restore intestinal motility.

## Graphical abstract

Slow‐transit constipation (STC) is a disabling motility disorder with unclear smooth‐muscle mechanisms. Spatial proteomic analysis of STC patient colon reveals both the central pathogenic role of smooth muscle cells (SMCs) in STC and novel regulators of intestinal motility, BIN1 and ALDH1B1.

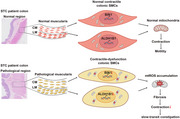

## 1 Introduction

Slow‐transit constipation (STC) is a subtype of functional constipation characterized by delayed colonic transit time, abdominal bloating, and infrequent defecation [1, 2, 3]. Although the pathophysiology of STC is not fully understood, it is known to involve abnormalities in the enteric nervous system (ENS) and a loss of interstitial cells of Cajal (ICC), which induce consequential alterations in smooth muscle structure and function [4, 5, 6]. However, the specific role of smooth muscle itself—particularly the regulatory mechanisms governing its structure and metabolism—remains insufficiently elucidated.

Intestinal motility relies fundamentally on the coordinated contraction of smooth muscle cells (SMCs), a process intricately linked to cytoskeletal dynamics and adequate cellular energy supply [7, 8]. Dysfunctions in these areas could directly impair contractile force and transit. Bridging integrator 1 (BIN1), also known as Amphiphysin‐2, is a multifunctional protein pivotal for membrane remodeling, trafficking, and cytoskeleton dynamics in a wide range of tissues [9, 10]. Its critical roles in neuronal synaptic function and in the transverse tubule (T‐tubule) formation and excitation‐contraction coupling of striated muscle are well‐established, with mutations linked to centronuclear myopathies (CNM) [11, 12, 13, 14, 15]. Despite these advances, the role of BIN1 in the human enteric neuromuscular system remains poorly understood, especially in the context of health versus STC.

Concurrently, cellular metabolism governed by mitochondrial enzymes provides the essential adenosine triphosphate (ATP) required for sustained muscle contractions [16, 17]. Aldehyde dehydrogenase 1 family member B1 (ALDH1B1) is considered a mitochondrial enzyme, which participates in aldehyde detoxification and retinoid metabolism, influencing cellular redox state and energy homeostasis [18, 19, 20, 21]. Although ALDH1B1 is expressed in the gut [18], a direct link between its function and impaired intestinal motility associated with STC remains unexamined.

Here, we demonstrate that the protein levels of BIN1 and ALDH1B1 are downregulated in STC patients, particularly in colonic SMCs localized to fibrotic foci. In vitro, targeted deletion of BIN1 or ALDH1B1 in HCoSMCs induced spontaneous fibrosis and led to decreased cell contraction. Mechanistically, BIN1 or ALDH1B1 downregulation compromised intestinal motility by damaging mitochondrial function, increasing reactive oxygen species (ROS) levels, and promoting extracellular matrix (ECM) deposition and fibrosis. This study establishes a smooth‐muscle‐centric view of STC and evaluates BIN1 and ALDH1B1 as candidate regulators of colonic motility.

## 2 Results

### 2.1 Spatial Proteomic Architecture of the Colonic Muscular Layer in STC Patients

**FIGURE 1:**
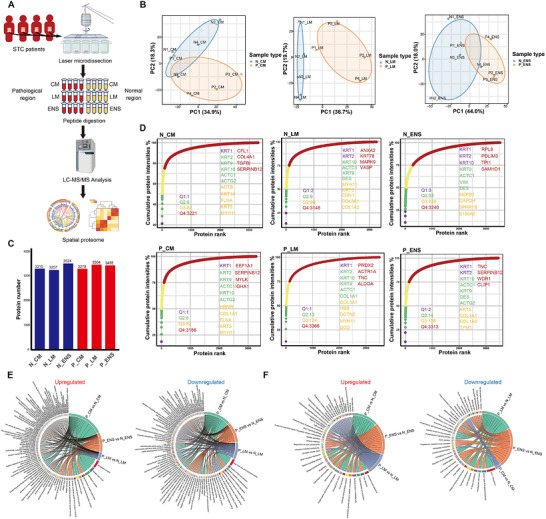
Spatial proteome profiled distinct protein signatures in region‐resolved human colon tissues. (A) Schematic of the experimental workflow of the sample collection, spatial proteome, and bioinformatics analysis to analyze the three regions of human colon tissues based on anatomy structure in the study: circular muscle (CM), Longitudinal muscle (LM), and enteric nervous system (ENS). Dissected samples by Laser microdissection from each structure were pooled, digested, and analyzed by liquid chromatography‐tandem mass spectrometry (LC‐MS/MS). (B) Principal coordinates analysis (PCA) of the proteome profile in the CM, LM, and ENS of colon tissues with pathological region and normal region from slow transit constipation (STC) patients. (C) Total number (as a bar graph) of identified protein groups for each region. (D) Cumulative protein abundances for the three structures of colon tissues with pathological region and normal region from STC patients and the total number of proteins constituting the quantiles (Q1: 25%, Q2: 5%, Q3: 75%, Q4: 100%). (E) Circos plot showing the Gene Ontology (GO) terms enriched in significantly upregulated and downregulated proteins of CM, LM, and ENS of colon tissues with pathological region and normal region from STC patients. (F) Circos plot showing the Kyoto Encyclopedia of Genes and Genomes (KEGG) pathways enriched in significantly upregulated and downregulated proteins of CM, LM, and ENS of colon tissues with pathological region and normal region from STC patients.

Intestinal motility dysfunction induces digestive system diseases such as constipation, especially STC [1, 22, 23]. To study the underlying molecular mechanisms in STC pathophysiology, four pairs of colon samples from STC patients, including three precise anatomical regions (CM, LM, ENS), were obtained for protein identification (Figure 1A,B). In total, 3524 proteins were identified in the four regions (Figure 1C). Subsequently, proteins in the different regions (CM, LM, ENS) of colon samples from STC patients in normal and pathological groups were divided into four parts according to the proportion of protein abundance (quantile Q1: 25%, Q2: 50%, Q3: 75%, Q4: 100%) to identify the protein with specifically high abundance in different regions (Figure 1D). For the normal groups, results showed that most of the highly expressed proteins (in Q1 or Q2) in different regions were keratins (KRT1, KRT2, KRT9, and KRT10), actins (ACTA1 and ACTG2), Desmin (DES), and vimentin (VIM), which were the skeleton and regulators of the muscle or nerve cells (Figure 1D). Other keratins (KRT5, KRT14, and KRT78), actin (ACTB), muscle myosin (MYH11), contraction regulation associated (FLNA, CFL1, VASP, CNN1, PDLIM3), collagens (COL1A2, COL3A1, and COL4A1), transforming growth factor (TGFBI), metabolism related (GAPDH and TPI1), calcium signal mediated (S100A6 and ANXA2), a component of ribosomes (RPL8), inflammation related (SAMHD1, HSPB1, and MAPK9), barrier associated (SPRR1B), were relatively lowly expressed (in Q3 or Q4) in the three regions of colon tissues (Figure 1D). For the pathological groups, collagen abundance was consistently higher and localized primarily to Q2 or Q3 compared to normal groups (Figure 1D), indicating that the colonic muscle layer was prone to fibrosis. The Cirplot diagram showed that the proteins associated with the extracellular matrix (ECM) were upregulated in three regions, especially in the CM (Figure 1E,F). In addition, proteins associated with the inflammatory response induced by pathogens in three regions were also upregulated (Figure 1F). Among downregulated proteins, those associated with energy metabolism were downregulated markedly in the CM, indicating that mitochondrial function of SMCs in the CM was severely damaged (Figure 1E). Proteins associated with the regulation of the actin cytoskeleton in the LM were downregulated, indicating serious intestinal smooth muscle damage (Figure 1F). These results suggest that cytoskeletal and metabolic alterations may contribute to impaired intestinal motility in STC

### 2.2 ENS Injury Impairs Intestinal Motility in STC Patients

The ENS is the core regulator of intestinal motility, autonomously regulating it by releasing various neurotransmitters [24, 25, 26, 27]. ENS injury directly leads to intestinal motility dysfunction [6, 28]. Spatial proteomics showed there were 205 differentially expressed proteins (DEPs), including 44 upregulated and 161 downregulated proteins, in the pathological ENS region (P_ENS) compared with the normal ENS region (N_ENS) in STC patients (Figure S1A). Next, we validated our proteomic discovery by IF staining and Western blotting (Figure S1B–E), which confirmed the decreased expression of CADM2 and KCTD12 in P_ENS. To assess the changes in ENS of STC patients, we further analyzed spatial proteomic data comparing P_ENS and N_ENS. As expected, a range of biological processes and signaling pathways associated with the immune response, the endoplasmic reticulum‐associated degradation (ERAD) pathway, sphingolipid metabolism, and the synaptic vesicle cycle were significantly enriched (Figure S1F,G). The enrichment of downregulated proteins in these processes and pathways suggests that ENS damage occurs in STC patients.

### 2.3 BIN1 Downregulation Induces Mitochondrial Dysfunction, Resulting in Impaired SMC Contraction

**FIGURE 2:**
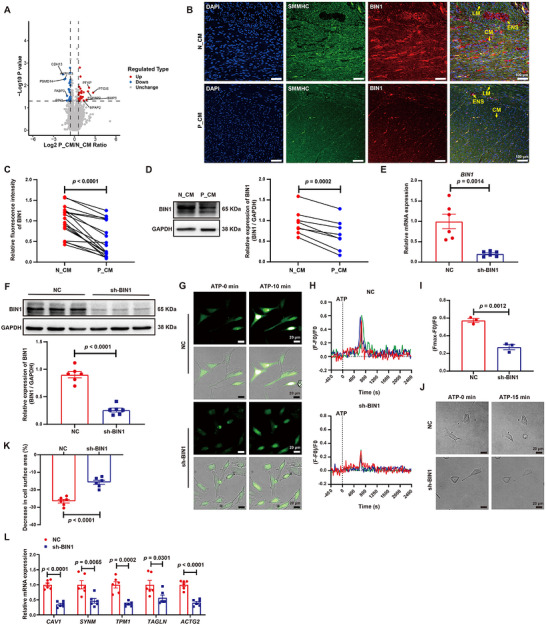
Downregulation of BIN1 in circular muscle inhibited smooth muscle cell contraction. Data are expressed as mean ± SEM. Data from in vitro assays are representative of at least three independent experiments. Statistical significance was analyzed using the paired two‐tailed t‐test (D, E) or the unpaired two‐tailed t‐test (F, G, J, L, M). Differences were considered statistically significant at *p* < 0.05. (A) Volcano plots visualized the differentially expressed proteins (DEPs) between pathological region and normal region of CM in colon tissues from STC patients. DEPs (*p* value < 0.05 and P_CM/N_CM Ratio > 1.5 or < 2/3) were highlighted in red (upregulated in P_CM group) and blue (downregulated in P_CM group). (B) Immunofluorescence images of SMMHC and BIN1 in colon tissues with pathological region and normal region from STC patients (scale bar: 100 µm). Yellow arrows pointed CM, LM, and ENS structure regions of the colon tissues. (C) Statistical graphs showing the fluorescence intensity of BIN1 between the pathological region and normal region of CM in colon tissues from STC patients (n = 18). (D) Western blot showing the protein expression of BIN1 between pathological region and normal region of CM in colon tissues from STC patients (n = 8). (E) qRT‐PCR showing the mRNA expression of BIN1 in HCoSMCs from NC group and sh‐BIN1 group (n = 6). (F) Western blot showing the protein expression of BIN1 in HCoSMCs from NC group and sh‐BIN1 group (n = 6). (G) Fluo‐4 AM (2 µM) staining of HCoSMCs for testing the intracellular Ca^2+^ concentration ([Ca^2+^]i). A higher fluorescence intensity indicated a higher [Ca^2+^]i. The representative pictures showed the 0‐ and 10‐min results after treatment with ATP (10 µM). (H) Statistical graphs showing the fluorescence intensity change during the photography period. Pictures were taken every 20 s. The fluorescence intensity change was expressed as the difference between the current fluorescence intensity F and the baseline fluorescence intensity F0 divided by the value of the baseline fluorescence intensity [(F‐F0)/F0] (n = 3). (I) Statistical graphs showing the comparison of maximum fluorescence intensity between NC group and sh‐BIN1 group (n = 3). (J) Pictures showing the change of cell surface area during the photography period. The representative pictures showed the 0‐ and 15‐min results after treatment with ATP (10 µM). (K) Statistical graphs showing the change rate of cell surface area after treatment with ATP (n = 6). Cell contraction was determined by the changes in the planar surface area. The percent decrease in surface area was calculated as [(the surface area of the cell after ATP–the surface area of the cell before ATP)/the surface area before ATP] × 100% using ImageJ software. (L) qRT‐PCR showing the mRNA expression of the smooth muscle contraction‐related genes in HCoSMCs from NC group and sh‐BIN1 group (n = 6).

While ENS integrity is significant for intestinal motility, effective peristalsis ultimately relies on the SMC contraction [4, 24]. SMC contraction is closely related to the cytoskeletal dynamics [8, 29]. Therefore, we examined the role of BIN1 (a multifunctional protein involved in cytoskeletal dynamics [9]) in intestinal motility. In spatial proteomics, there were 51 DEPs, including 25 upregulated and 26 downregulated proteins, in the pathological CM region (P_CM) compared with the normal CM region (N_CM) in STC patients (Figure 2A). To validate the observed decrease in FABP3 and BIN1 expression in P_CM, we performed IF staining and Western blotting, both of which corroborated the proteomic findings (Figure 2B–D and Figure S2A–C). In vivo experiments revealed that the expression levels of the BIN1 gene and protein were significantly reduced in the circular muscle of STC mice (Figure S3A–D). Moreover, after AAV‐induced knockdown of Bin1 in colonic smooth muscle, the mice exhibited increased susceptibility to loperamide‐induced STC (Figure S3E–H). In vitro, we constructed stable HCoSMCs by using lentivirus‐mediated BIN1 knockdown (sh‐BIN1) (Figure 2E,F). To investigate the role of BIN1 in SMC contraction, BIN1‐knockdown HCoSMCs were treated with ATP (10 µM) during culture. Besides serving as an essential energy currency for cellular metabolism, ATP also acts as an extracellular messenger, regulating smooth muscle contraction through activation of distinct cell surface receptors of the P2 receptor family [30, 31]. As expected, Fluo‐4 AM (2 µM) staining showed changes in intracellular fluorescence intensity after ATP treatment were lower in the sh‐BIN1 group than in the NC group (Figure 2G–I). Consistently, compared with the NC group, the sh‐BIN1 group exhibited a significantly smaller reduction in cell surface area after ATP treatment (Figure 2J,K). Moreover, the expression of cell contraction‐related genes was significantly reduced after BIN1 knockdown (Figure 2L). Meanwhile, AAV‐induced knockdown of Bin1 in colonic smooth muscle further suppressed the expression of contraction‐related genes in the circular muscle cells of STC mice (Figure S3I). Importantly, in vitro experiments demonstrated that overexpression of BIN1 effectively rescued the impaired contractile function of HCoSMCs caused by BIN1 knockdown (Figure S4A–E). The above results indicate that BIN1 downregulation leads to impaired SMC contraction.

**FIGURE 3:**
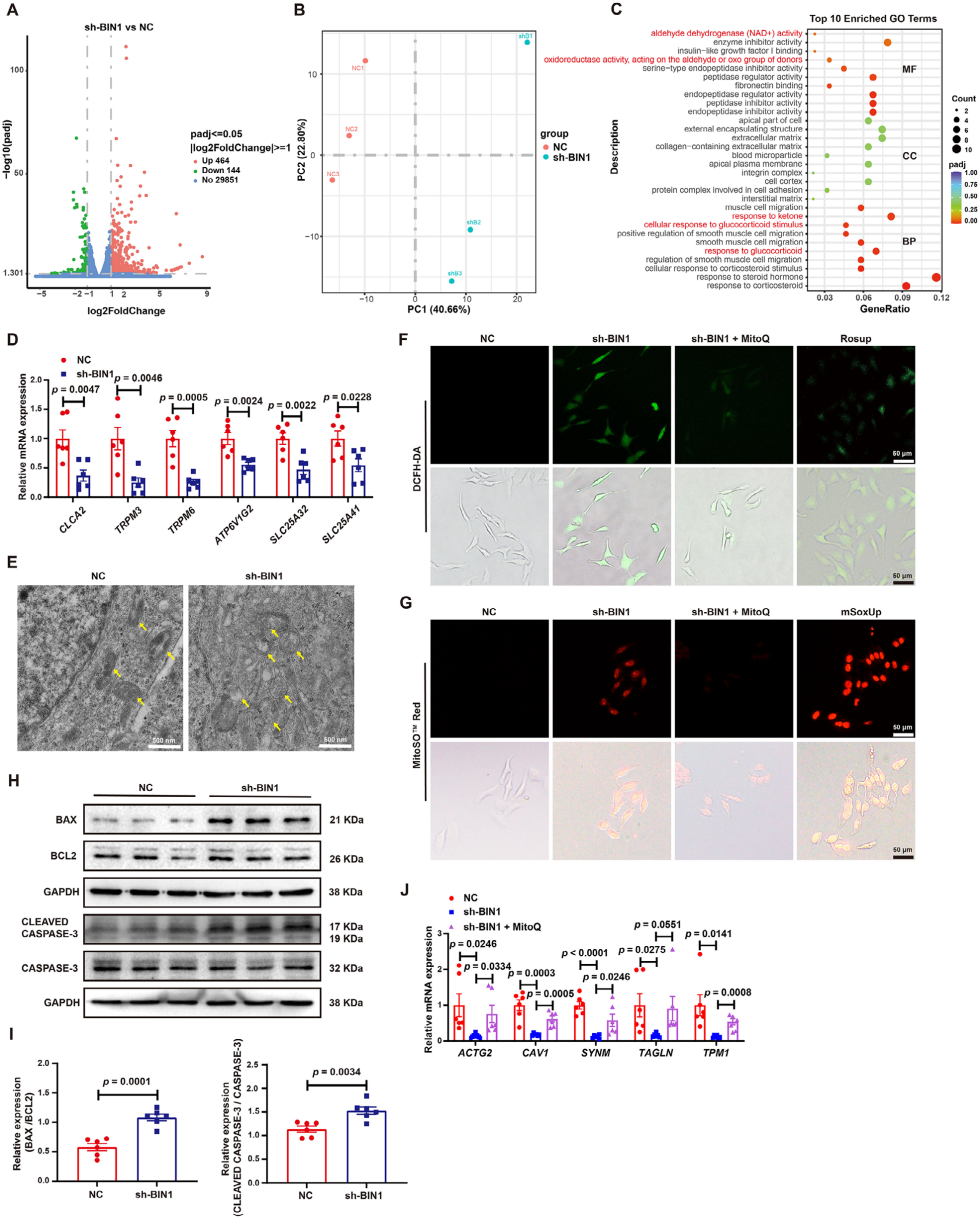
Loss of BIN1 disrupted mitochondrial homeostasis and activated the mitochondrial apoptotic pathway. Data are expressed as mean ± SEM. Data from in vitro assays are representative of at least three independent experiments. Statistical analyses were performed by the unpaired two‐tailed t‐test. Differences were considered statistically significant at *p* < 0.05. (A) The volcano plot visualized the differentially expressed genes (DEGs) of HCoSMCs from NC group and sh‐BIN1 group. DEGs (padj ≤ 0.05 and |log2(FoldChange)| ≥ 1) were highlighted in red (upregulated in sh‐BIN1 group) and green (downregulated in sh‐BIN1 group). (B) PCA of RNA‑seq data from HCoSMCs in NC and sh‑BIN1 groups (n = 3). (C) Dot plot showing the top 10 enriched terms in the Molecular Function (MF), Cellular Component (CC), and Biological Process (BP) categories from the GO analysis. The enriched terms related to mitochondria were highlighted in red. (D) qRT‐PCR showing the mRNA expression of the mitochondrial function‐related genes in HCoSMC from NC group and sh‐BIN1 group (n = 6). (E) The transmission electron microscopy observation in HCoSMCs from NC group and sh‐BIN1 group (scale bar: 500 nm). The yellow arrow indicated mitochondria. (F) DCFH‐DA (10 µM) staining showing the content of ROS in HCoSMCs from NC, sh‐BIN1, sh‐BIN1 + MitoQ, and Rosup groups (scale bar: 50 µm). Rosup (50 µg/mL) served as a positive control. MitoQ is a mitochondria‐targeted antioxidant. (G) MitoSO Red (5 µM) staining showing the mitochondrial ROS Levels in HCoSMCs from NC, sh‐BIN1, sh‐BIN1 + MitoQ, and mSoxUp groups (scale bar: 50 µm). 1X mSoxUp served as a positive control. MitoQ is a mitochondria‐targeted antioxidant. (H) Western blot showing the expression of proteins related to mitochondrial apoptotic pathway in HCoSMCs from NC group and sh‐BIN1 group (n = 6). (I) Statistical graphs showing the comparison on the expression of proteins related to mitochondrial apoptotic pathway between NC group and sh‐BIN1group (n = 6). (J) qRT‐PCR showing the mRNA expression of the smooth muscle contraction‐related genes in HCoSMCs from NC, sh‐BIN1, and sh‐BIN1 + MitoQ groups (n = 6).

To determine how BIN1 downregulation impaired SMC contraction, we performed RNA sequencing (RNA‐seq) on HCoSMCs from the NC and sh‐BIN1 groups. Overall, 464 upregulated and 144 downregulated genes were identified (Figure 3A,B). Further analysis revealed that a range of mitochondrial function‐related molecular functions [such as aldehyde dehydrogenase (NAD+) activity and oxidoreductase activity] and biological processes (such as response to ketone and glucocorticoid) were significantly enriched (Figure 3C). We speculate that BIN1 downregulation affects mitochondrial function. As speculated, the expression of mitochondrial function‐related genes was significantly reduced after BIN1 knockdown (Figure 3D). Consistently, transmission electron microscopy (TEM) observed that BIN1 knockdown resulted in damaged mitochondrial architecture, including ablation of cristae, in HCoSMCs (Figure 3E). DCFH‐DA is a fluorogenic dye that is used to detect total ROS generation [32, 33]. To further investigate mitochondrial function in BIN1‐knockdown HCoSMCs, we evaluated mitochondrial ROS (mtROS) by MitoSO Red staining. A significant rise in mtROS levels was observed in the HCoSMCs with BIN1 knockdown (Figure 3F,G). Furthermore, Western blotting demonstrated that the elevated protein activations of BAX, CLEAVED CASPASE‐3 in the sh‐BIN1 group were significantly higher than those in the NC group (Figure 3H,I). Results indicate that BIN1 knockdown disrupts mitochondrial function. Moreover, we used MitoQ (a mitochondria‐targeted antioxidant) to scavenge mtROS in BIN1 knockdown cells and found that mtROS removal significantly rescued the contractile dysfunction caused by BIN1 downregulation (Figure 3J). Taken together, BIN1 downregulation coincided with mitochondrial defects and reduced SMC contraction.

### 2.4 ALDH1B1 Downregulation Inhibits SMC Contraction by Inducing Mitophagy

**FIGURE 4:**
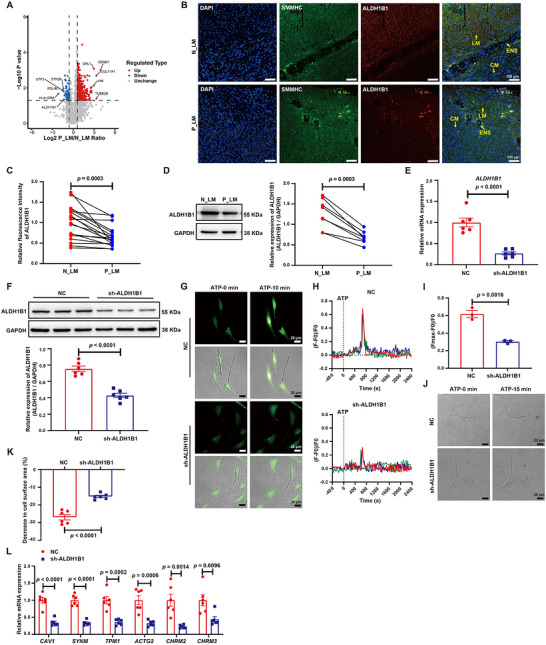
Downregulation of ALDH1B1 in the longitudinal muscle inhibited smooth muscle cell contraction. Data are expressed as mean ± SEM. Data from in vitro assays are representative of at least three independent experiments. Statistical significance was analyzed using the paired two‐tailed t‐test (D, E) or the unpaired two‐tailed t‐test (F, G, J, L, M). Differences were considered statistically significant at *p* < 0.05. (A) Volcano plots visualized the DEPs between pathological region and normal region of LM in colon tissues from STC patients. DEPs (*p* value < 0.05 and P_LM/N_LM Ratio > 1.5 or < 2/3) were highlighted in red (upregulated in P_LM group) and blue (downregulated in P_LM group). (B) Immunofluorescence images of SMMHC and ALDH1B1 in colon tissues with pathological region and normal region from STC patients (scale bar: 100 µm). Yellow arrows pointed CM, LM, and ENS structure regions of colon tissues. (C) Statistical graphs showing the fluorescence intensity of ALDH1B1 between pathological region and normal region of LM in colon tissues from STC patients (n = 18). (D) Western blot showing the protein expression of ALDH1B1 between pathological region and normal region of LM in colon tissues from STC patients (n = 8). (E) qRT‐PCR showing the mRNA expression of ALDH1B1 in HCoSMCs from NC group and sh‐ALDH1B1 group (n = 6). (F) Western blot showing the protein expression of ALDH1B1 in HCoSMCs from NC group and sh‐ALDH1B1 group (n = 6). (G) Fluo‐4 AM (2 µM) staining of HCoSMCs for testing the intracellular Ca^2+^ concentration ([Ca^2+^]i). A higher fluorescence intensity indicated a higher [Ca^2+^]i. The representative pictures showed the 0‐ and 10‐min results after treatment with ATP (10 µM). (H) Statistical graphs showing the fluorescence intensity change during the photography period. Pictures were taken every 20 s. The fluorescence intensity change was expressed as the difference between the current fluorescence intensity F and the baseline fluorescence intensity F0 divided by the value of the baseline fluorescence intensity [(F‐F0)/F0] (n = 3). (I) Statistical graphs showing the comparison of maximum fluorescence intensity between NC group and ALDH1B1 group (n = 3). (J) Pictures showing the change of cell surface area during the photography period. The representative pictures showed the 0‐ and 15‐min results after treatment with ATP (10 µM). (K) Statistical graphs showing the change rate of cell surface area after treatment with ATP (n = 6). Cell contraction was determined by the changes in the planar surface area. The percent decrease in surface area was calculated as [(the surface area of cell after ATP–the surface area of the cell before ATP)/the surface area before ATP] × 100% using ImageJ software. (L) qRT‐PCR showing the mRNA expression of the smooth muscle contraction‐related genes in HCoSMCs from NC group and sh‐ALDH1B1 group (n = 6).

In addition to cytoskeletal dynamics, SMC contraction also relies on energy supply [7, 34, 35]. ALDH1B1 is a mitochondrial enzyme, which participates in energy homeostasis [21]. In spatial proteomics, there were 339 DEPs, including 265 upregulated and 74 downregulated proteins, in the pathological LM region (P_LM) compared with the normal LM region (N_LM) in STC patients (Figure 4A). Among these DEPs, ALDH1B1 belonged to one of the downregulated proteins. Subsequent validation of our proteomic findings via IF staining and Western blotting confirmed reduced expression of ALDH1B1 and SLMAP in P_LM (Figure 4B–D and Figure S5A–C). In vivo studies demonstrated a marked decrease in both ALDH1B1 gene and protein expression within the longitudinal muscle of STC mice (Figure S6A–D). Moreover, knockdown of Aldh1b1 in colonic smooth muscle mediated by AAV vectors heightened the susceptibility of mice to loperamide‐induced STC (Figure S6E–H). Using lentivirus‐mediated ALDH1B1 knockdown (sh‐ALDH1B1), we established stable HCoSMCs in vitro (Figure 4E,F). Next, we assessed SMC contraction by stimulating ALDH1B1‐knockdown HCoSMCs with ATP (10 µM) during culture. Fluo‐4 AM staining revealed a less pronounced increase in intracellular fluorescence intensity following ATP treatment in the sh‐ALDH1B1 group compared to the NC group (Figure 4G–I). Similarly, the contraction (decrease in cell surface area) triggered by ATP was significantly less in the sh‐ALDH1B1 group relative to the NC group (Figure 4J,K). Additionally, ALDH1B1 silencing significantly suppressed the expression of genes involved in cell contraction (Figure 4L). Furthermore, knockdown of Aldh1b1 in colonic smooth muscle via AAV vectors led to a further reduction in the expression of contraction‐associated genes within the longitudinal muscle cells of STC mice (Figure S6I). Notably, in vitro studies confirmed that overexpression of ALDH1B1 significantly restored the contractile dysfunction in HCoSMCs that was induced by ALDH1B1 knockdown (Figure S7A–E). Collectively, these data demonstrate that reduced ALDH1B1 expression causes a deficit in SMC contraction.

**FIGURE 5:**
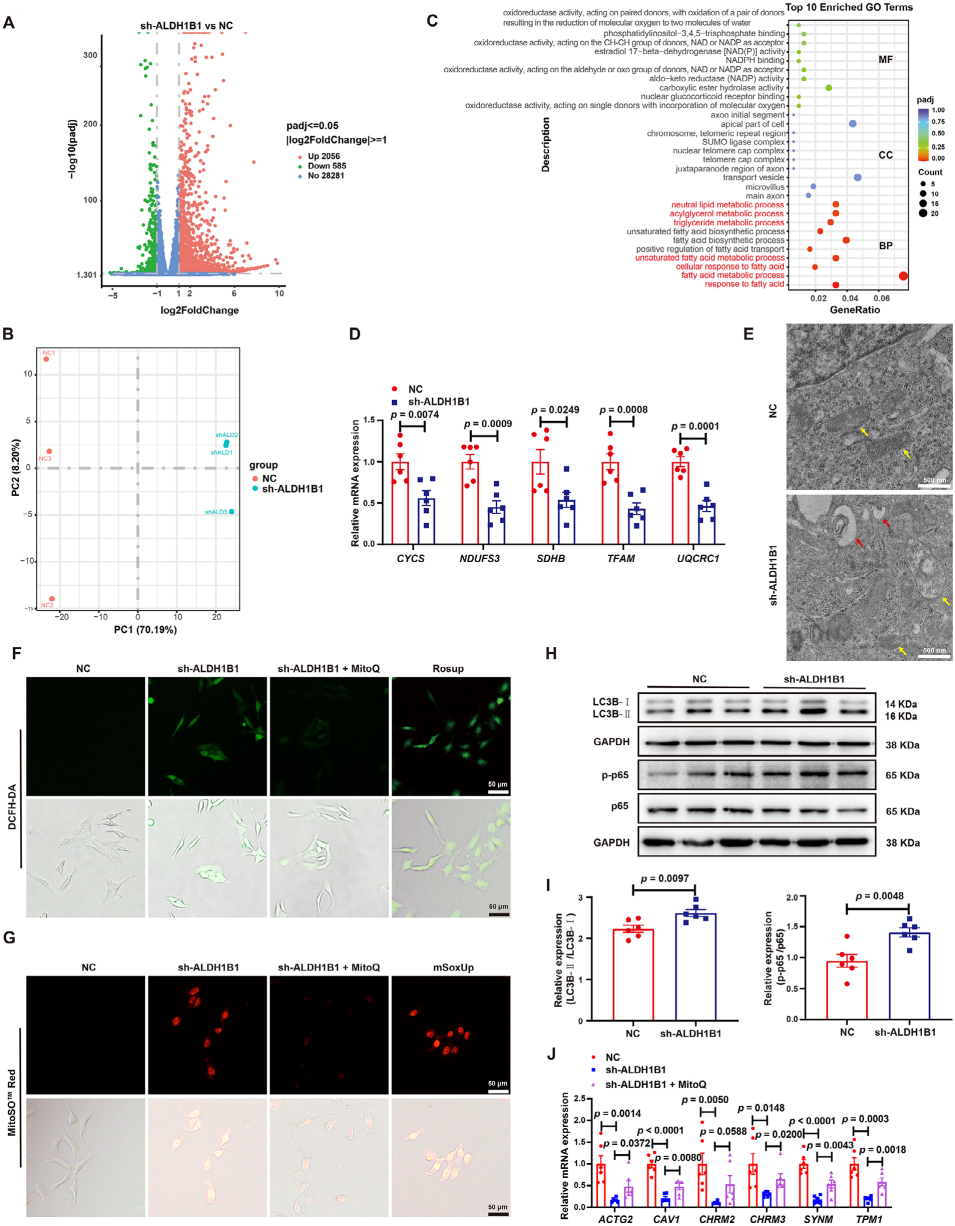
ALDH1B1 deficiency impaired mitochondrial homeostasis, triggering aberrant autophagy and pro‐inflammatory activation. Data are expressed as mean ± SEM. Data from in vitro assays are representative of at least three independent experiments. Statistical analyses were performed by the unpaired two‐tailed t‐test. Differences were considered statistically significant at *p* < 0.05. (A) The volcano plot visualized the differentially expressed genes (DEGs) of HCoSMC from NC group and sh‐ALDH1B1 group. DEGs (padj ≤ 0.05 and |log2(FoldChange)| ≥ 1) were highlighted in red (upregulated in sh‐ALDH1B1 group) and green (downregulated in sh‐ALDH1B1 group). (B) PCA of RNA‑seq data from HCoSMCs in NC and sh‑ALDH1B1 groups (n = 3). (C) Dot plot showing the top 10 enriched terms in the MF, CC, and BP categories from the GO analysis. The enriched terms related to mitochondria were highlighted in red. (D) qRT‐PCR showing the mRNA expression of the mitochondrial function‐related genes in HCoSMCs from NC group and sh‐ALDH1B1 group (n = 6). (E) The transmission electron microscopy observation in HCoSMCs from NC group and sh‐ALDH1B1 group (scale bar: 500 nm). The yellow arrow indicated mitochondria. The red arrow indicated the autophagy process. (F) DCFH‐DA (10 µM) staining showing the content of ROS in HCoSMCs from NC, sh‐ALDH1B1, sh‐ALDH1B1 + MitoQ and Rosup groups (scale bar: 50 µm). Rosup (50 µg/mL) served as a positive control. MitoQ is a mitochondria‐targeted antioxidant. (G) MitoSO Red (5 µM) staining showing the mitochondrial ROS Levels in HCoSMCs from NC, sh‐ALDH1B1, sh‐ALDH1B1 + MitoQ and mSoxUp groups (scale bar: 50 µm). 1X mSoxUp served as a positive control. MitoQ is a mitochondria‐targeted antioxidant. (H) Western blot showing the protein expression of LC3B and p65 in HCoSMCs from NC group and sh‐ALDH1B1 group (n = 6). (I) Statistical graphs showing the comparison on the protein expression of Figure H above between NC group and sh‐ALDH1B1 group (n = 6). (J) qRT‐PCR showing the mRNA expression of the smooth muscle contraction‐related genes in HCoSMCs from NC, sh‐ALDH1B1, and sh‐ALDH1B1 + MitoQ groups (n = 6).

To elucidate the underlying mechanism of impaired SMC contraction following ALDH1B1 knockdown, RNA‐seq analysis was performed on HCoSMCs from the NC and sh‐ALDH1B1 groups. Altogether, the analysis revealed 2056 upregulated genes and 585 downregulated genes (Figure 5A,B). Downregulated genes were found to be significantly enriched in a variety of fatty acid metabolism‐related biological processes (Figure 5C). Meanwhile, ALDH1B1 depletion led to a significant reduction in mitochondrial function‐related gene expression (Figure 5D). Based on these observations, we postulate that downregulating ALDH1B1 disrupts mitochondrial function. TEM findings demonstrated that knocking down ALDH1B1 in HCoSMCs led to mitochondrial cristae ablation and mitophagy (Figure 5E). Besides, the DCFH‐DA and MitoSO Red assay showed a significant rise in mtROS levels following ALDH1B1 knockdown (Figure 5F,G). Notably, Western blot quantification confirmed a significant upregulation in LC3B and p‐p65 activation in sh‐ALDH1B1 groups versus NC groups (Figure 5H,I). These findings collectively demonstrate that ALDH1B1 knockdown induces mitophagy and activates inflammatory signaling pathways. Moreover, we used MitoQ to scavenge mtROS in ALDH1B1 knockdown cells and found that mtROS removal significantly rescued the contractile dysfunction caused by ALDH1B1 downregulation (Figure 5J). In summary, loss of ALDH1B1 impairs SMC contraction function through triggering mitophagy.

### 2.5 Loss of BIN1 or ALDH1B1 in HCoSMCs Is Associated With Fibrosis‐Related Transcriptional Programs

**FIGURE 6:**
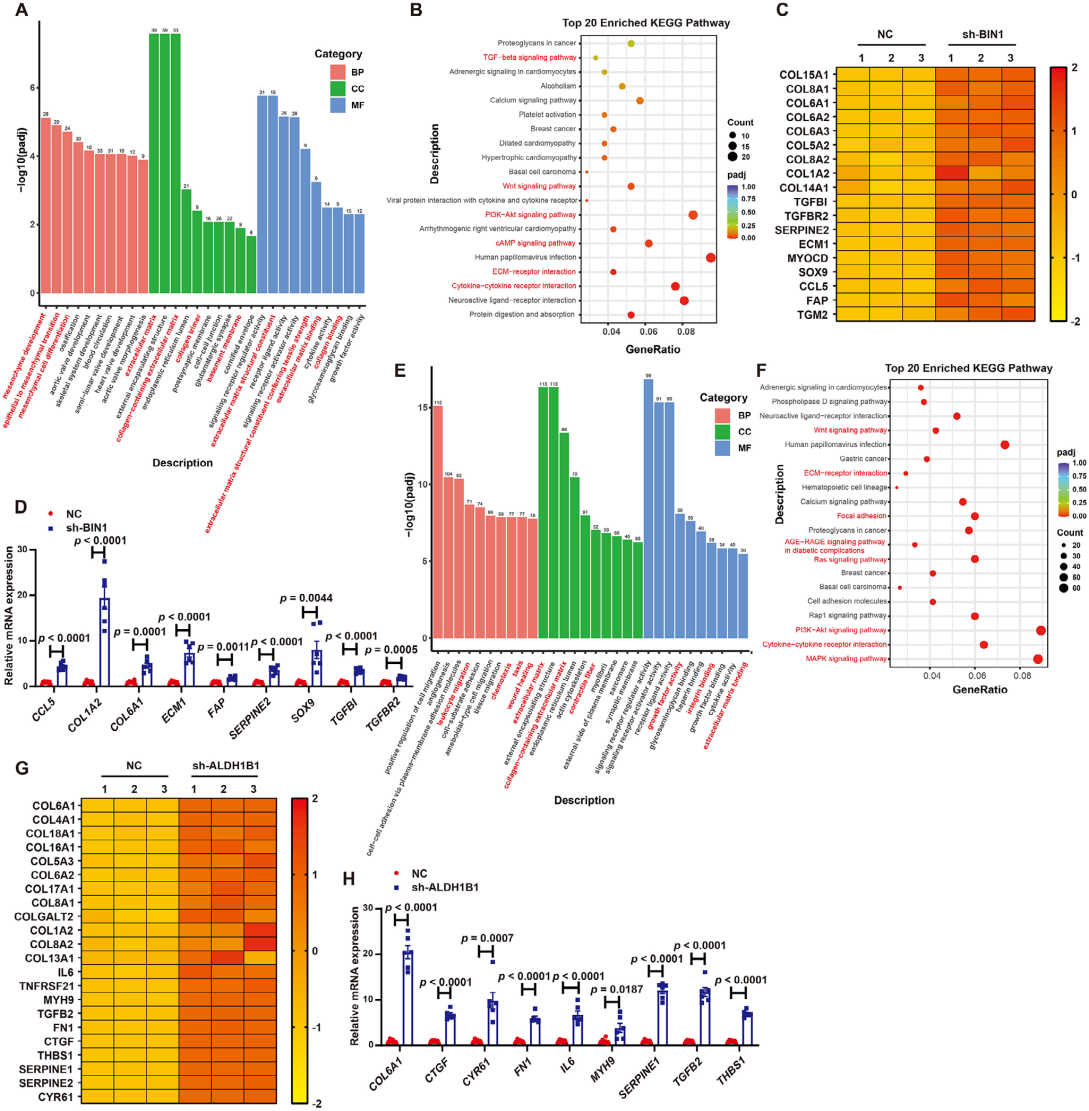
Knockdown of BIN1 or ALDH1B1 induces fibrosis‐related transcriptional programs in HCoSMCs. Data are expressed as mean ± SEM. Data from in vitro assays are representative of at least three independent experiments. Statistical analyses were performed by the unpaired two‐tailed t‐test. Differences were considered statistically significant at *p* < 0.05. (A) Bar graph showing the top 10 enriched terms between NC group and sh‐BIN1 group in the MF, CC, and BP categories from the GO analysis. The enriched terms related to profibrotic response were highlighted in red. (B) Dot plot showing the top 20 enriched KEGG pathway between NC group and sh‐BIN1 group. The enriched terms related to profibrotic response were highlighted in red. (C) Heatmap showing the expression of profibrotic response‐related genes in HCoSMCs from NC group and sh‐BIN1 group (n = 3). (D) qRT‐PCR showing the mRNA expression of the profibrotic response‐related genes in HCoSMCs from NC group and sh‐BIN1 group (n = 6). (E) Bar graph showing the top 10 enriched terms between NC group and sh‐ALDH1B1 group in the MF, CC, and BP categories from the GO analysis. The enriched terms related to profibrotic response were highlighted in red. (F) Dot plot showing the top 20 enriched KEGG pathway between NC group and sh‐ALDH1B1 group. The enriched terms related to profibrotic response were highlighted in red. (G) Heatmap showing the expression of profibrotic response‐related genes in HCoSMCs from NC group and sh‐ALDH1B1 group (n = 3). (H) qRT‐PCR showing the mRNA expression of the profibrotic response‐related genes in HCoSMCs from NC group and sh‐ALDH1B1 group (n = 6).

Beyond mitochondrial impairment, RNA‐seq identified a series of pro‐fibrotic molecular signatures triggered by persistent BIN1/ALDH1B1 deficiency. Gene Ontology (GO) enrichment analysis highlighted upregulated‐gene enrichment of biological functions related to fibrosis, including biological processes (e.g., mesenchyme development, epithelial to mesenchymal transition), cellular components (e.g., extracellular matrix, collagen trimer, basement membrane), and molecular functions (e.g., extracellular matrix binding, collagen binding) in BIN1 knockdown HCoSMCs (Figure 6A). Similarly, Kyoto Encyclopedia of Genes and Genomes (KEGG) enrichment analysis showed that upregulated genes were significantly enriched in pro‐fibrotic pathways following BIN1 depletion, such as cytokine‐cytokine receptor interaction, ECM‐receptor interaction, cAMP, PI3K‐Akt, Wnt and TGF‐β signaling (Figure 6B). Therefore, we focused on all differentially expressed fibrotic‐related genes (Figure 6C). Note that multiple collagen genes were upregulated in sh‐BIN1 groups versus NC groups (Figure 6C). ECM deposition is an important marker of fibrosis [36, 37, 38, 39]. qRT‐PCR validated the findings of the RNA‐seq analysis (Figure 6D). These results indicate that BIN1 deficiency in HCoSMCs is associated with a pro‐fibrotic gene expression program. In HCoSMCs with ALDH1B1 knockdown, GO enrichment analysis of upregulated genes revealed enrichment in fibrosis‐associated terms across biological processes (e.g., leukocyte migration, chemotaxis, wound healing), cellular components (e.g., extracellular matrix, contractile fiber), and molecular functions (e.g., growth factor activity, integrin binding, extracellular matrix binding) (Figure 6E). Meanwhile, upon ALDH1B1 depletion, upregulated genes were significantly enriched for pro‐fibrotic KEGG pathways, such as cytokine‐cytokine receptor interaction, ECM‐receptor interaction, MAPK, PI3K‐Akt, AGE−RAGE, and Wnt signaling (Figure 6F). Notably, the expression of fibrosis‐linked genes, specifically multiple collagen genes, was elevated in sh‐ALDH1B1 groups relative to NC groups (Figure 6G). qRT‐PCR confirmed the heatmap results (Figure 6H). These data suggest that ALDH1B1 is associated with increased expression of fibrosis‐related genes. It is noteworthy that both restoring the expression of the knockdown gene (BIN1 or ALDH1B1) and scavenging the mtROS induced by gene knockdown can suppress the expression of pro‐fibrotic genes in the knockdown cells (Figure S8A–D). Collectively, these data demonstrate that both BIN1 and ALDH1B1 suppress the fibrotic progression of HCoSMCs by maintaining mitochondrial ROS homeostasis.

### 2.6 Colonic Fibrosis Is a Major Contributor to Intestinal Dysmotility in STC Patients

**FIGURE 7:**
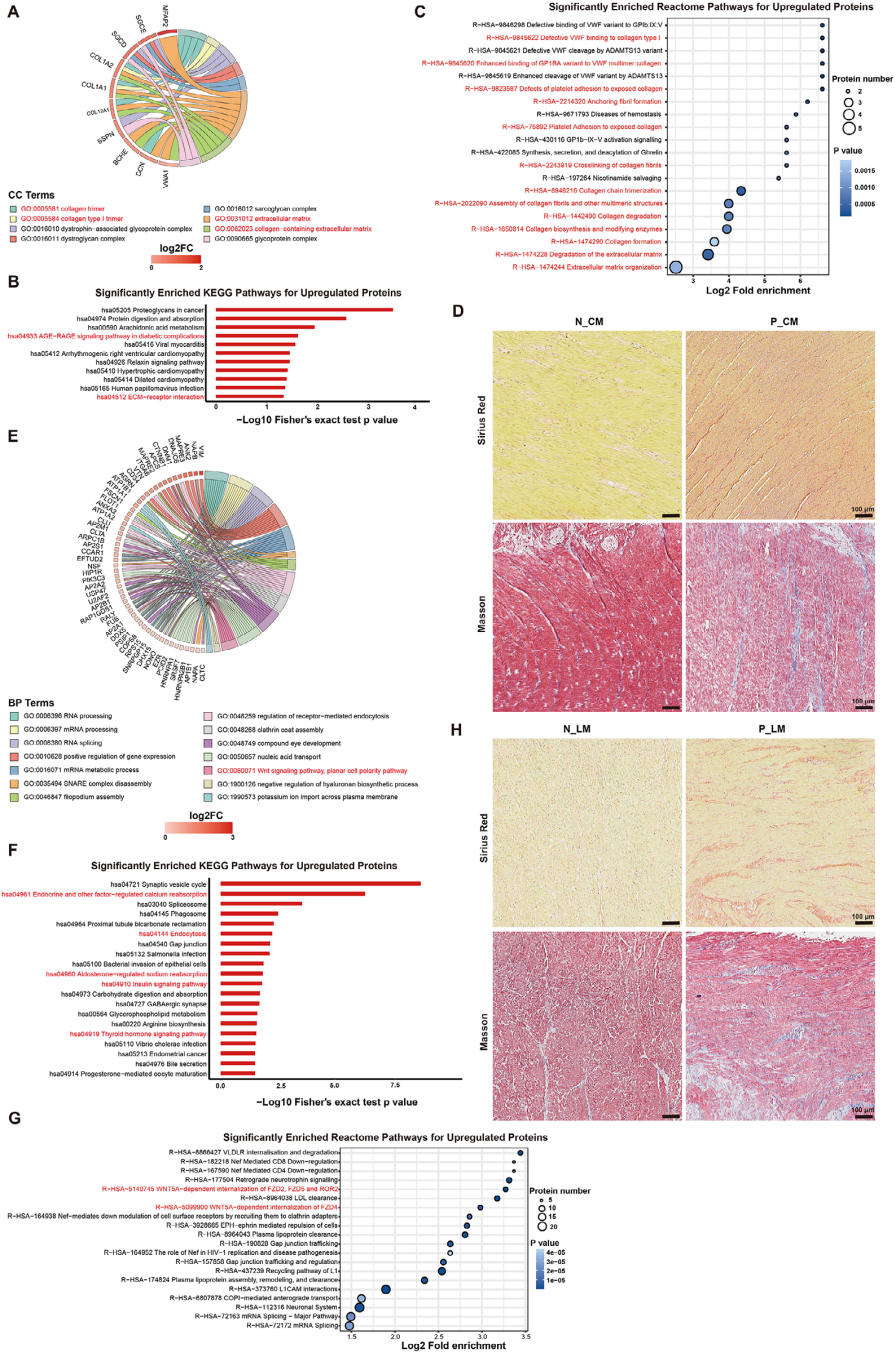
Fibrosis‐related enrichment and collagen deposition in STC colon. (A) Circle plot showing the selected GOCC terms for the enrichment of upregulated protein clusters in CM. The enriched terms related to profibrotic response were highlighted in red. (B) Bar graph showing the significantly enriched KEGG pathways for upregulated proteins in CM. The enriched terms related to profibrotic response were highlighted in red. (C) Dot plot showing the significantly enriched Reactome pathways for upregulated proteins in CM. The enriched terms related to profibrotic response were highlighted in red. (D) Representative images of CM sections stained with Sirius Red and Masson (scale bar: 100 µm). (E) Circle plot showing the selected GOBP terms for the enrichment of upregulated protein clusters in LM. The enriched terms related to profibrotic response were highlighted in red. (F) Bar graph showing the significantly enriched KEGG pathways for upregulated proteins in LM. The enriched terms related to profibrotic response were highlighted in red. (G) Dot plot showing the significantly enriched Reactome pathways for upregulated proteins in LM. The enriched terms related to profibrotic response were highlighted in red. (H) Representative images of LM sections stained with Sirius Red and Masson (scale bar: 100 µm).

To determine whether downregulation of BIN1 and ALDH1B1 also contributes to fibrosis in the colon of STC patients, we conducted further validation using spatial proteomic datasets and patient‐derived intestinal sections. We performed GOCC, KEGG and Reactome analysis to determine function enrichment of upregulated proteins in CM of STC patients. Consistent with previous findings, a series of fibrosis‐related cellular components were significantly enriched, such as collagen trimer, extracellular matrix (Figure 7A). More importantly, multiple collagen deposition‐related signaling pathways were observed, including ECM‐receptor interaction, Crosslinking of collagen fibrils, Collagen chain trimerization, Collagen biosynthesis and modifying enzymes, Collagen formation, and Extracellular matrix organization (Figure 7B,C). In addition, Sirius Red and Masson staining confirmed fibrosis development in the CM of STC patients (Figure 7D). In the same way, we performed GOBP, KEGG, and Reactome analysis to determine function enrichment of upregulated proteins in LM of STC patients. The analysis revealed a series of fibrosis‐related biological processes and signaling pathways, including Wnt signaling, Endocrine and other factor‐regulated calcium reabsorption, Endocytosis, Aldosterone‐regulated sodium reabsorption, Insulin signaling, and Thyroid hormone signaling (Figure 7E–G). Similarly, Sirius Red and Masson staining demonstrated the development of fibrosis in the LM of STC patients (Figure 7H). Meanwhile, qRT‐PCR demonstrated an upregulation of pro‐fibrotic genes in the colonic smooth muscle cells of STC mice. AAV‐mediated knockdown of Bin1 or Aldh1b1 further upregulated these pro‐fibrotic genes (Figure S9A,B). Consistently, the Sirius Red and Masson staining also confirmed these results (Figure S9C–F). In summary, downregulation of BIN1 and ALDH1B1 in colonic smooth muscle contributes to colonic fibrosis with subsequent impairment of intestinal motility in STC patients.

## 3 Discussion

This study identified BIN1 and ALDH1B1 as novel regulators of intestinal motility. We revealed significant BIN1 and ALDH1B1 downregulation in colonic smooth muscle of STC patients. Mechanistically, BIN1 deficiency in CM significantly promoted the progression of colonic fibrosis via activating the mitochondrial apoptotic pathway, thus inhibiting SMC contraction. Concurrently, ALDH1B1 deficiency in LM accelerated colonic fibrosis development by inducing both mitophagy and activating the inflammatory response, thereby compromising SMC contraction. These results provide important insights into the role of BIN1 and ALDH1B1 in intestinal motility.

BIN1 is a ubiquitously expressed protein that plays a critical role in endocytosis, trafficking, and cytoskeletal dynamics [9, 40]. In skeletal and cardiac muscles, BIN1 is essential for the maturation and maintenance of the T‐tubules, a crucial cellular structure for muscle contraction [9, 41]. For example, CNM is associated with mutations in the gene that encodes BIN1 [14]. BIN1 downregulation contributes to abnormal cardiac contraction and elevates the risk of malignant arrhythmias prior to heart failure [42]. While BIN1 has been implicated in skeletal and cardiac muscles injury, its role in colonic smooth muscle remains underexplored. Notably, our results showed that BIN1 was crucial for maintaining SMC contraction function in the colon. Compared with the N_CM, BIN1 expression in the P_CM of STC patients was significantly decreased. SMC‐specific BIN1 deletion promoted mitochondrial apoptotic pathway activation and ECM deposition, exacerbating colonic fibrosis and inhibiting SMC contraction—consistent with reports showing BIN1 knockdown worsens ventricular contractility [43]. These data collectively establish BIN1 as the regulator of intestinal motility.

ALDH1B1 is a mitochondrial enzyme, and previous studies have found that it is highly expressed mainly in the liver, pancreas, small intestine, colon, and lung tissues of humans and mice [18, 44]. In tumor research, ALDH1B1 is considered a new marker of cancer progression, such as colorectal cancer and pancreatic cancer [45, 46]. Significantly, a recent study has found that ALDH1B1 in hepatocytes protects against nonalcoholic steatohepatitis (NASH) by maintaining ROS levels [47]. Similarly, our results demonstrated that downregulation of ALDH1B1 in HCoSMCs promoted mtROS accumulation and consequent NF‐κB (p65) pathway activation. Moreover, TEM observed the appearance of mitophagosomes. These pathological changes synergistically drove colonic fibrosis, thus inhibiting intestinal motility. In colon tissues from STC patients, ALDH1B1 expression was significantly reduced in the LM where fibrosis was present. Collectively, these findings implicate ALDH1B1 as a critical contributor to intestinal motility regulation.

From a clinical perspective, our study reveals a novel pathological mechanism in STC, highlighting the pivotal role of SMCs themselves, rather than the ENS or ICCs [5, 48, 49]. Deficiencies in BIN1 and ALDH1B1 disrupt mitochondrial integrity and trigger interconnected cascades leading to intestinal dysmotility. Specifically, loss of BIN1 in CM activates the mitochondrial apoptotic pathway, elevating ROS levels, inducing ECM deposition and fibrosis, and suppressing of SMC contractility. Concurrently, ALDH1B1 deficiency in LM impairs mitochondrial turnover via aberrant mitophagy, further increasing ROS, amplifying inflammatory signals, and exacerbating ECM‐driven fibrosis and SMC dysfunction. These defects converge to amplify shared pathological processes—ROS overload, fibrotic remodeling, and impaired SMC contraction—that collectively culminate in compromised intestinal motility [50, 51, 52]. The convergence of BIN1 and ALDH1B1 deficiencies in driving fibrosis and intestinal dysmotility suggests that these pathways may represent promising therapeutic entry points for STC. Nevertheless, it must be noted that there is essentially no research on targeted drug interventions for both at present. Some laboratories have designed small molecules targeting ALDH1B1, such as IGUANAs [18], but none have entered clinical practice. This absence of pharmacological tools underscores both the novelty of our findings and an urgent need for future drug development efforts.

This study has several limitations. One potential limitation is the possible development of resistance via upregulation of P‐glycoprotein (P‐gp) during the 14‐day administration of loperamide, as loperamide is a known substrate of P‐gp. Chronic exposure to P‐gp substrates can theoretically induce P‐gp expression, which might reduce the drug's efficacy over time. However, in this study, we confirmed that loperamide remained pharmacologically effective throughout the treatment period by verifying the successful establishment of the STC model at the end of the 14‐day regimen. Multiple functional parameters (reduced fecal pellet number, decreased fecal water content, prolonged colonic transit time, and extended whole gut transit time) were significantly altered compared to the control group, indicating that the inhibitory effect on intestinal motility was sustained. Therefore, while we acknowledge the theoretical risk of P‐gp‐mediated resistance, our experimental data suggest that any potential upregulation did not substantially compromise the pharmacological action of loperamide within this 14‐day timeframe. Future studies with longer treatment durations or direct measurement of P‐gp expression levels would be needed to further address this issue.

In addition, although we have in vivo evidence using an AAV‐mediated knockdown combined with a loperamide‐induced STC mouse model, this approach does not fully replace more rigorous genetic models. Tissue‐wide mCherry reporter distribution was not systematically examined. Therefore, although qRT‐PCR and western blotting confirmed efficient knockdown in the isolated colonic smooth muscle layers, the AAV2/9‐SM22α system should be interpreted as a smooth muscle‐enriched rather than strictly colon‐specific knockdown approach. Future studies using inducible colonic smooth‑muscle‑specific conditional knockout models will be needed to establish definitive tissue‑ and cell‑type‑specific causality, and to determine whether loss of Bin1 or Aldh1b1 is sufficient to drive STC‑related pathology independently. Moreover, while MitoQ treatment and gene re‐expression experiments support mitochondrial ROS as an important downstream mediator of the profibrotic and contractile phenotypes, the precise molecular mechanisms connecting BIN1/ALDH1B1 deficiency to mitochondrial dysfunction and fibrosis remain incompletely defined. Finally, the discovery cohort for spatial proteomics was modest in size, which limited the ability to perform subgroup analyses. In addition, the cohort included patients with various secondary causes of intestinal motility disorders to increase the sample size for the subsequent validation experiments. While this approach reflected real‐world clinical scenarios, the resulting heterogeneity may confound the analysis of these validation experiments, particularly given that the limited sample size of these experiments precluded stratification by etiology. Therefore, larger clinically stratified cohorts, with stratification by specific etiology, will be needed to further validate the relevance of these findings in human STC and to provide more precise mechanistic insights.

In conclusion, the present study demonstrated that reduced BIN1 and ALDH1B1 expression may contribute to impaired intestinal motility by disrupting mitochondrial function, increasing ROS levels, and promoting fibrotic remodeling. This study not only elucidates BIN1 and ALDH1B1 as novel regulators of intestinal motility but also highlights the central role of SMCs in the pathogenesis of STC.

## 4 Materials and Methods

### 4.1 Ethics Statement

All human samples and clinical data used in this study were collected after obtaining informed consent from participants and were approved by the Ethics Committee of Shandong Provincial Third Hospital (KYLL‐2025150). The information of STC patients is listed in Table S1.

### 4.2 Animals

Male C57BL/6J mice (4–5 weeks, 16–18 g) were obtained from Beijing Vital River Laboratory Animal Technology Co., Ltd. All mice were housed in a temperature‐controlled environment (24°C ± 2°C) under a 12/12‐h light/dark cycle with ad libitum access to food and water. All animal experiments were approved by the Ethical Committee of the School of Basic Medical Sciences, Shandong University (Shandong, China, ECSBMSSDU2024‐2‐41).

### 4.3 Adeno‐Associated Virus (AAV2/9) Short Hairpin RNA (shRNA) for Gene Knockdown

pAAV‐SM22ap‐MCS‐mCherry‐miR30shRNA (NC)‐WPRE (Control), pAAV‐SM22ap‐MCS‐mCherry‐miR30shRNA (Aldh1b1)‐WPRE (sh‐Aldh1b1), and pAAV‐SM22ap‐MCS‐mCherry‐miR30shRNA (Bin1)‐WPRE (sh‐Bin1) were obtained from Shanghai Obio Technology (Group) Corp., Ltd. (Shanghai, China). For Aldh1b1 knockdown, 4–5‐week‐old mice received a tail vein injection of AAV2/9 (sh‐Aldh1b1) or control virus at a concentration of 5 × 10^11^ vg per mouse, administered two weeks before the establishment of the STC mouse model. The same procedure was followed for Bin1 knockdown using AAV2/9 (sh‐Bin1). Knockdown efficiency was validated by qRT‐PCR and Western blot. The target sequence for mouse Aldh1b1 was: 5’‐GTGTGGGTGAACACCTATAAC‐3’. The target sequence for mouse Bin1 was: 5’‐GTGTAGGTTTCTATGTCAACA‐3’. The NC sequence was: 5’‐GAAGTCGTGAGAAGTAGAA‐3’.

### 4.4 STC Model

Two weeks after tail vein injection of AAV2/9 into SPF C57BL/6J mice, loperamide (10 mg/kg body weight) was orally administered for 14 days to establish the STC mouse model. Subsequently, fecal output, pellet water content, whole gut transit time, and colonic transit time were monitored. Loperamide was obtained from Shanghai Yuanye Bio‐Technology Co., Ltd. (Shanghai, China).

### 4.5 Measurement of Fecal Output and Pellet Water Content

The number of fecal pellets expelled by each mouse was recorded every 30 min over a 6‐h period to assess gastrointestinal motility. The average hourly fecal pellet output for each mouse was then calculated to enable comparisons between mice. Subsequently, one fresh fecal pellet from each mouse was collected into a separate sterile microcentrifuge tube. After measuring the wet weight, each sample was oven‐dried for 48 h to obtain the dry weight. The fecal water content for each mouse was calculated as the difference between the wet and dry weights of this representative fecal pellet.

### 4.6 Determination of Whole Gut Transit Time

At the endpoint (e.g., day 14) of each animal experiment, mice fasted overnight while water was provided. Subsequently, all mice received an oral gavage of 250 µL of a 5% carmine red (Sigma, Germany, C1022) suspension (prepared in 0.5% sodium carboxymethyl cellulose). Immediately after gavage, each mouse was transferred to a clean, empty individual cage with free access to food and water. The whole gut transit time was recorded as the time interval between the gavage and the expulsion of the first fecal pellet stained red.

### 4.7 Determination of Colonic Transit Time

At the endpoint (e.g., day 15) of each animal experiment, mice were fasted overnight while water was provided. Then, a 3‐mm‐diameter glass bead maintained at 37°C was gently inserted into the colon via the anus to a depth of 2 cm. Immediately after insertion, each mouse was transferred to a clean, empty individual cage with free access to food and water. The colonic transit time was recorded as the interval between bead insertion and the expulsion of the bead. The experiment was terminated once the bead was expelled. After sacrificing the mice, colon tissue samples from STC mice were dissected under microscopic guidance to remove the mucosal layer, separating the longitudinal muscle and circular muscle.

### 4.8 Cell Culture

Human colonic smooth muscle cell (HCoSMC) used in this study was obtained from ScienCell Research Laboratories (Carlsbad, CA, USA, #2940). Cells were cultured in smooth muscle cell culture medium (SMCM, #1101).

### 4.9 Generation of Knockdown Cells

Lentiviral vectors were purchased from Shanghai Genechem Co., Ltd. (Shanghai, China). ALDH1B1‐knockdown (sh‐ALDH1B1) HCoSMCs and BIN1‐knockdown (sh‐BIN1) HCoSMCs were generated using lentiviral transfection methods following the manufacturer's instructions. In brief, cells were transduced with a lentiviral plasmid expressing shRNA targeting ALDH1B1, BIN1 or a non‐targeting control (negative control, NC). Cells were then subjected to puromycin selection. Knockdown efficiency was validated by qRT‐PCR and Western blot. The target sequence for human ALDH1B1 was: 5’‐GAATCCATCTACAATGAGTTT‐3’. The target sequence for human BIN1 was: 5’‐GTGCGTCCAGAATTTCAACAA‐3’. The NC sequence was: 5’‐TTCTCCGAACGTGTCACGT‐3’.

### 4.10 Establishment of Overexpression Cells

The lentiviral overexpression vectors pcSLenti‐EF1‐Puro‐CMV‐ALDH1B1‐WPRE (ALDH1B1‐OE) and pcSLenti‐EF1‐Puro‐CMV‐BIN1‐WPRE (BIN1‐OE) were obtained from Shanghai Obio Technology (Group) Corp., Ltd. (Shanghai, China). To generate the ALDH1B1‐KD + OE and BIN1‐KD + OE HCoSMCs, the respective knockdown cells were transduced with the corresponding vectors. Stable overexpressing cells were then established through puromycin selection.

### 4.11 Cell Contractility Assay

ALDH1B1 knockdown HCoSMCs and BIN1 knockdown HCoSMCs were imaged using an Opera Phenix high‐content screening system (PerkinElmer, USA). Cells were treated with ATP (10 µM, MCE, New Jersey, USA, HY‐B2176). Pictures were taken every 30 s. Cell contraction was determined by the changes in the planar surface area as previously reported [53]. The perimeters of individual cell with clearly defined borders were outlined, and the percent decrease in surface area was calculated as [(the surface area of cell after ATP–the surface area of the cell before ATP)/the surface area before ATP] × 100% using ImageJ software.

### 4.12 Ca^2+^ Imaging

ALDH1B1 knockdown HCoSMCs and BIN1 knockdown HCoSMCs were washed with DMEM and then covered with a sufficient amount of Fluo4 AM (2 µM, Beyotime, Shanghai, China, S1060) in DMEM at 37°C for 30 min to measure changes in cytosolic Ca^2+^ ion concentration. Cells were imaged using an Opera Phenix high‐content screening system (PerkinElmer, USA). Images were immediately stored on a high‐speed hard drive. The ratio of fluorescence intensity changes excited at 488 nm and collected at 512–520 nm was computed as previously reported [54, 55]. During the photography period, both the NC group and shRNA group cells were treated with ATP (10 µM, MCE, HY‐B2176). Pictures were taken every 20 s. The fluorescence intensity change was expressed as the difference between the current fluorescence intensity F and the baseline fluorescence intensity F0 divided by the value of the baseline fluorescence intensity [(F‐F0)/F0].

### 4.13 Transmission Electron Microscopy (TEM)

The cells were centrifuged into a cell cluster and fixed in 2.5% glutaraldehyde fixative. The subsequent process was conducted as previously described [56]. Transmission electron microscope (Thermo Fisher Scientific, USA, Talos F200C) was used to observe the morphological features of mitochondria.

### 4.14 Reactive Oxygen Species (ROS) Detection

Cellular ROS levels were measured by the usage of ROS Assay Kit (Beyotime, S0033S). Dichloro‐dihydro‐fluorescein diacetate (DCFH‐DA) was diluted in a serum‐free DMEM medium at a ratio of 1:1000 to a final concentration of 10 µM. The cell culture medium was removed, and an appropriate volume of diluted DCFH‐DA was added. 0.5 mL of diluted DCFH‐DA was usually added to per well of the 24‐well plates. HCoSMCs were incubated in an incubator at 37°C for 30 min and washed 3 times with serum‐free DMEM medium to fully remove the DCFH‐DA that did not enter into cells. Rosup was a mixture used as a positive control. Cells in the positive control group were pretreated with Rosup (50 µg/mL) for 20 min before incubation with DCFH‐DA. A fluorescence microscope (Nikon, Japan) was required to observe its fluorescence intensity.

### 4.15 Mitochondrial ROS Detection

Mitochondrial ROS levels were measured by the usage of Mitochondrial Superoxide Assay Kit (Beyotime, S0061S). MitoSO Red was diluted in a serum‐free DMEM medium at a ratio of 1:1000 to a final concentration of 5 µM. The cell culture medium was removed, and an appropriate volume of diluted MitoSO Red was added. 0.5 mL of diluted MitoSO Red was usually added to per well of the 24‐well plates. HCoSMCs were incubated in an incubator at 37°C for 30 min and washed 3 times with serum‐free DMEM medium to fully remove the MitoSO Red that did not enter into cells. mSoxUp was a mixture used as a positive control. Cells in the positive control group were pretreated with mSoxUp (1X) for 4 h before incubation with MitoSO Red. A fluorescence microscope (Nikon, Japan) was required to observe its fluorescence intensity.

### 4.16 Mitochondrial ROS Scavenging Assay

To further validate the effect of gene knockdown on oxidative stress, the experiment was conducted using established HCoSMCs with gene knockdown. Following treatment with MitoQ (500 nM, MCE, USA, HY‐100116A) for 24 h, the cells were subjected to staining with DCFH‐DA and MitoSOX Red to detect total ROS and mitochondrial ROS, respectively.

### 4.17 Western Blotting Analysis

Following surgery, colon tissue samples from STC patients were dissected under microscopic guidance to remove the mucosal layer, separating the longitudinal muscle and circular muscle. The myenteric plexus remained attached to the circular muscle layer. Proteins were extracted from HCoSMCs, mouse or human samples using RIPA lysis buffer (Solarbio, Beijing, China, R0020), supplemented with protein phosphatase inhibitor (Solarbio, P1260) and protease inhibitor mixture (Solarbio, P6730) under ice bath conditions. The protein concentration was determined by a Bicinchoninic Acid Protein Assay kit (Beyotime, P0010). Then, the same amounts of protein were separated by SDS‐PAGE and transferred onto polyvinylidene difluoride (PVDF) membranes (Millipore, Massachusetts, USA, IPVH00010). The PVDF membranes were blocked with 5% nonfat dry milk and incubated overnight with the corresponding primary antibodies at 4°C according to experimental requirements. The following primary antibodies were used: GAPDH (60004‐1‐Ig, Proteintech, 1:10000), KCTD12 (15523‐1‐AP, Proteintech, 1:1000), BIN1 (14647‐1‐AP, Proteintech, 1:1000), FABP3 (10676‐1‐AP, Proteintech, 1:1000), ALDH1B1 (15560‐1‐AP, Proteintech, 1:1000), SLMAP (ab243383, Abcam, 1:1000), CADM2 (PA5‐106550, Invitrogen, 1:1000), BAX (#2772, Cell Signaling Technology, 1:1000), BCL2 (#3498, Cell Signaling Technology, 1:1000), Cleaved Caspase‐3 (#9661, Cell Signaling Technology, 1:1000), Caspase‐3 (#9662, Cell Signaling Technology, 1:1000), LC3B (#43566, Cell Signaling Technology, 1:1000), p‐p65 (#3033, Cell Signaling Technology, 1:1000), p65 (#8242, Cell Signaling Technology, 1:1000).Then, the membranes were washed with Tris Buffered Saline with Tween 20 (TBST) and incubated with corresponding secondary antibodies including HRP‐conjugated Goat Anti‐Rabbit IgG(H+L) (SA00001‐2, Proteintech, 1:5000) or HRP‐conjugated Goat Anti‐Mouse IgG(H+L) (SA00001‐1, Proteintech, 1:5000). Protein bands was visualized using the ChemiDoc XRS system and Image Lab Software following covered with BeyoECL PLUS (Beyotime, P0018S) and the protein expression was quantified with ChemiDoc XRS system and Image Lab Software (Bio‐Rad, Hercules, USA).

### 4.18 Real Time Quantitative PCR (qRT‐PCR) Analysis

Total RNA from HCoSMCs and mouse samples was extracted with SparkZol Reagent (Sparkjade, Shandong, China, AC0101‐B). Further, cDNA was obtained according to the protocol of PrimeScript RT Reagent Kit (Takara, Japan, RR037A), and qRT‐PCR was conducted using the UltraSYBR Mixture (CWBIO, Beijing, China, CW0957H) on the Applied Biosystems StepOne Real‐Time PCR System (Thermo Fisher Scientific, USA). Gene normalization was analyzed by comparison with the expression of its control gene (GAPDH), and the fold changes were analyzed using the 2^−ΔΔCt^ method. The primers used were listed in Table S2.

### 4.19 Immunofluorescence, Masson, and Sirius Red Staining

Paraffin slides were deparaffinized and rehydrated with xylene and graded alcohol. They were immersed in 0.01 M sodium citrate buffer (pH 6.0) in a microwaved for antigen retrieval. After natural cooling, sections were incubated with endogenous peroxidase blocker for 30 min at room temperature to quench endogenous peroxidase activity. 0.5% Triton X‐100 was used to penetrate the cell membrane. Then, sections were blocked with goat serum (ZSGB‐BIO, Beijing, China) for 1 h. After blocking, the sections were incubated with the following primary antibodies: KCTD12 (15523‐1‐AP, Proteintech, 1:200), BIN1 (14647‐1‐AP, Proteintech, 1:200), FABP3 (10676‐1‐AP, Proteintech, 1:200), ALDH1B1 (15560‐1‐AP, Proteintech, 1:200), SLMAP (ab243383, Abcam, 1:200), CADM2 (PA5‐106550, Invitrogen, 1:200), SMMHC (60222‐1‐Ig, Proteintech, 1:200), TUBB3 (66375‐1‐Ig, Proteintech, 1:200). After incubating the primary antibodies overnight, secondary antibodies were incubated after washing for three times. The sections were then washed and stained with 4′,6‐diamidino‐2‐phenylindole (DAPI). Images were pictured using a fluorescence microscope (Nikon, Japan). Masson's trichrome staining (Solarbio, G1340) and Sirius Red staining (Solarbio, G1472) were performed according to the manufacturer's protocols. Images were observed using a Nikon microscope.

### 4.20 RNA Sequencing (RNA‐seq) Analysis

HCoSMCs were collected through routine digestion and subsequently subjected to RNA‐seq. HCoSMCs from NC and shRNA (sh‐ALDH1B1 and sh‐BIN1) groups, encapsulated in SparkZol Reagent, were forwarded to Novogene Co., Ltd. (Beijing, China) for further processing. There were 4 groups of samples, divided into 2 projects, which used different NC groups. Three samples, derived from wells of separate 6‐well plates, were prepared for each group. The Strand‐specific library was constructed according to the manufacturer's instructions. Transcriptome sequencing was performed using the Illumina NovaSeq 6000 platform. DESeq2 algorithms were used for identifying differentially expressed genes (DEGs), with the selection criteria of |log2(FoldChange)| > 1 and an adjusted *p*‐value (*p*adj) < 0.05.

### 4.21 Laser‐Capture Microdissection (LCM)

Sample was obtained from surgeries and divided into small pieces and then washed with PBS. The tissue was transferred in O.C.T. Compound (Solarbio, China) and snap frozen. The O.C.T.‐embedded tissue was sectioned at 10 µm on a CM1950 cryostat (Leica) and mounted onto PEN membrane‐coated glass slides. The tissue sections were stained with hematoxylin and eosin (H&E). Then, the sections were visualized on digital slide scanner SLIDEVIEW VS200 (OLYMPUS) at 20 × magnification. LCM procedure was completed on the Laser‐Capture Microdissection System PALM (Zeiss). The cutting parameters were optimized to minimize laser damage at first. Under the supervision of experienced pathologists, three pathological regions with well‐defined morphology characteristics—circular muscle (CM), longitudinal muscle (LM), and enteric nervous system (ENS)—were selected and dissected. The microdissected samples were collected with microtubes (Zeiss, 415190‐9201‐000) and stored at −80°C or digested for proteomic.

### 4.22 Peptide Digestion

The microdissected samples were resuspended with lysis buffer and were sonicated using a contactless high‐intensity ultrasonic processor (Scientz) and then were incubated at 95°C for 5 min. Peptide digestion was performed overnight at 37°C. The peptides solution was reduced with 5 mM dithiothreitol for 30 min at 56°C and alkylated with 11 mM iodoacetamide for 15 min at room temperature in darkness. Finally, the peptides were desalted with C18 ZipTips (Millipore) according to the manufacturer's instructions.

### 4.23 LC‐MS/MS Analysis

The tryptic peptides were dissolved in solvent A and directly loaded onto a custom‐packed reversed‐phase analytical column (15‐cm length, 100‐µm I.D.). Peptides were separated using a mobile phase consisting of solvent A (0.1% formic acid in water) and solvent B (0.1% formic acid and 80% acetonitrile in water) with the following gradient at a constant flow rate of 200 nL/min on a Vanquish Neo UPLC system (Thermo Fisher Scientific): 0 to 1.6 min, 4% to 22.5% B; 1.6 to 2.0 min, 22.5% to 35% B; 2.0 to 2.1 min, 35% to 35.1% B; 2.1 to 2.3 min, 35.1% B; 2.3 to 9.2 min, 35.1% to 35.2% B; 9.2 to 9.6 min, 35.2% to 55% B; 9.6 to 10.1 min, 55% to 99% B; 10.1 to 12.0 min, 99% B. The separated peptides were analyzed using a nano‐electrospray ion source coupled to an Orbitrap Astral mass spectrometer. An electrospray voltage of 1900 V was applied. Intact precursor ions were analyzed in the Orbitrap analyzer, and fragment ions were analyzed in the Astral analyzer. Full MS scans were performed over the range of 400–800 m/z with a resolution of 240,000. MS/MS scans were acquired with a fixed first mass of 150.0 m/z and a resolution of 80,000. Fragmentation was performed using higher‐energy collisional dissociation (HCD) with a normalized collision energy (NCE) of 25%. The automatic gain control (AGC) target was set to 800%, with a maximum injection time of 15 ms. The DIA data were processed using the DIA‐NN software (version 1.8). Tandem mass spectra were searched against the Homo_sapiens_9606_SP_20231220.fasta database (containing 20,429 entries), concatenated with a reverse decoy database. Trypsin/P was specified as the cleavage enzyme, allowing for up to 1 missed cleavage. The fixed modifications specified were excision of the N‐terminal methionine and carbamidomethylation on cysteine. The false discovery rate (FDR) was adjusted to < 1%.

### 4.24 Quantification and Statistical Analysis

Statistical analysis and visualization of the proteome were performed using Perseus software (2.0.7.0) and R Studio (4.1.0). In regional enriched protein cluster analysis, the ratio of the mean relative quantification values of each protein between two sample groups was used as the Fold Change (FC). To determine statistical significance, a t‐test was performed on the relative quantification values of each protein in the comparison groups. The resulting *p*‐value served as the significance indicator, with a significance threshold set at *p*‐value < 0.05. Significant proteins (*p* value < 0.05) with FC > 1.5 were deemed up‐regulated, while those with FC < 1/1.5 were deemed down‐regulated.

### 4.25 Statistical Analyses

All statistical analyses were performed using GraphPad Prism 9.0. Data are expressed as mean ± SEM. Normality was assessed with the Shapiro–Wilk test. Normally distributed data were analyzed by unpaired t‐tests (or paired t‐tests for paired human samples). Non‐normally distributed data were compared using the Mann–Whitney U test. Each in vitro experiment was replicated at least three times with independent biological samples. Each in vivo experiment was replicated at least six times with independent biological samples. Differences were considered statistically significant at *p* < 0.05.

## Author Contributions

J.B.L. and H.Z. performed the main experiments, analyzed the data, and wrote the original draft. W.Q., R.L., J.S., X.L., D.Z., and Q.L. helped to collect part of the original data. S.D., J.L., D.C., and H.Z. designed the study, revised the paper, and provided financial support. All authors read and approved the final manuscript.

## Funding

This study was supported by the National Natural Science Foundation of China (Nos. 32271172, 32471177, 81770542) and the Natural Science Foundation of Shandong Province of China (ZR2019MH119). This work was supported in part by the Jiangsu Specially‐Appointed Professor Program, the Anti‐tumor New Drug Rapid Translation Public Service Platform of Jiangsu Province (BM2023002), Joint Research Grant of Shandong Provincial Third Hospital and Cheeloo Medical College (LHXM2023ZD12003), Medical and Health Science and Technology Development Project of Shandong Province (202504011287) and Traditional Chinese Medicine Science and Technology Project of Shandong Province (M20252905).

## Conflicts of Interest

The authors declare no conflicts of interest.

## Supporting information




**Supporting File**: advs76104‐sup‐0001‐SuppMat.docx.

## Data Availability

The mass spectrometry proteomics data have been deposited to the ProteomeXchange [57] Consortium via the PRIDE [58] partner repository with the dataset identifier PXD069723 (Username: reviewer_pxd069723@ebi.ac.uk; Password: 4A5dy0Sle0ZP). Further information and requests for data can be addressed to the corresponding author, Jingxin Li (ljingxin@sdu.edu.cn).

## References

[1] A. E. Bharucha and B. E. Lacy , “Mechanisms, Evaluation, and Management of Chronic Constipation,” Gastroenterology 158, no. 5 (2020): 1232–1249, 10.1053/j.gastro.2019.12.034. 31945360 PMC7573977

[2] M. Camilleri , A. C. Ford , G. M. Mawe , et al., “Chronic Constipation,” Nature Reviews Disease Primers 3 (2017): 17095, 10.1038/nrdp.2017.95. 29239347

[3] F. Chi , W. Sun , C. Zhang , et al., “Single‐Cell Transcriptomics Reveals the Interaction between Fibroblasts and Activated Immune Cells: an Exploratory Bioinformatics Study of Pro‐Inflammatory Mechanisms in Slow Transit Constipation,” International Journal of Surgery 111, no. 6 (2025): 3767–3780, 10.1097/JS9.0000000000002415. 40265474 PMC12165498

[4] F. Daoud , J. Holmberg , A. Alajbegovic , et al., “Inducible Deletion of YAP and TAZ in Adult Mouse Smooth Muscle Causes Rapid and Lethal Colonic Pseudo‐Obstruction,” Cellular and Molecular Gastroenterology and Hepatology 11, no. 2 (2021): 623–637, 10.1016/j.jcmgh.2020.09.014. 32992050 PMC7806867

[5] Z. Gao , L. Fu , W. Bai , and J. Liang , “Protective Effects of Medicinal Plant‐Derived Metabolites on Slow Transit Constipation via the ENS‐ICC‐SMC Pathway,” Frontiers in Pharmacology 16 (2025): 1598806, 10.3389/fphar.2025.1598806. 40567379 PMC12188447

[6] M. J. Workman , M. M. Mahe , S. Trisno , et al., “Engineered human Pluripotent‐Stem‐Cell‐Derived Intestinal Tissues with a Functional Enteric Nervous System,” Nature Medicine 23, no. 1 (2017): 49–59, 10.1038/nm.4233. PMC5562951 27869805

[7] P. Hu , M. Liu , T. Wu , et al., “Calcium Dysregulation Disrupts Mitochondrial Homeostasis by Interfering AMPK/Drp1 Pathway to Aggravate Plaque Progression and Instability,” Theranostics 15, no. 15 (2025): 7567–7583, 10.7150/thno.112041. 40756371 PMC12315820

[8] M. Xin , E. M. Small , L. B. Sutherland , et al., “MicroRNAs miR‐143 and miR‐145 Modulate Cytoskeletal Dynamics and Responsiveness of Smooth Muscle Cells to Injury,” Genes & Development 23, no. 18 (2009): 2166–2178, 10.1101/gad.1842409. 19720868 PMC2751981

[9] Q. Giraud and J. Laporte , “Amphiphysin‐2 (BIN1) Functions and Defects in Cardiac and Skeletal Muscle,” Trends in Molecular Medicine 30, no. 6 (2024): 579–591, 10.1016/j.molmed.2024.02.005. 38514365

[10] L. Picas , C. André‐Arpin , F. Comunale , et al., “BIN1 regulates Actin‐Membrane Interactions during IRSp53‐Dependent Filopodia Formation,” Communications Biology 7, no. 1 (2024): 549, 10.1038/s42003-024-06168-8. 38724689 PMC11082164

[11] P. De Rossi , T. Nomura , R. J. Andrew , et al., “Neuronal BIN1 Regulates Presynaptic Neurotransmitter Release and Memory Consolidation,” Cell Reports 30, no. 10 (2020): 3520–3535, 10.1016/j.celrep.2020.02.026. 32160554 PMC7146643

[12] L. Al‐Qusairi and J. Laporte , “T‐Tubule Biogenesis and Triad Formation in Skeletal Muscle and Implication in human Diseases,” Skeletal Muscle 1, no. 1 (2011): 26, 10.1186/2044-5040-1-26. 21797990 PMC3156648

[13] A. De La Mata , S. Tajada , S. O'Dwyer , et al., “BIN1 Induces the Formation of T‐Tubules and Adult‐Like Ca^2+^ Release Units in Developing Cardiomyocytes,” Stem Cells 37, no. 1 (2019): 54–64, 10.1002/stem.2927. 30353632 PMC6312737

[14] C. Gineste , A. Simon , M. Braun , D. Reiss , and J. Laporte , “Tamoxifen Improves Muscle Structure and Function of Bin1‐ and Dnm2‐related Centronuclear Myopathies,” Brain 146, no. 7 (2023): 3029–3048, 10.1093/brain/awac489. 36562127

[15] Q. Giraud , C. Spiegelhalter , N. Messaddeq , and J. Laporte , “MTM1 overexpression Prevents and Reverts BIN1‐Related Centronuclear Myopathy,” Brain 146, no. 10 (2023): 4158–4173, 10.1093/brain/awad251. 37490306 PMC10545525

[16] J. Zhang , X. Wang , F. Wang , and X. Tang , “Xiangsha Liujunzi Decoction Improves Gastrointestinal Motility in Functional Dyspepsia with Spleen Deficiency Syndrome by Restoring Mitochondrial Quality Control Homeostasis,” Phytomedicine 105 (2022): 154374, 10.1016/j.phymed.2022.154374. 35963194

[17] Y. Suzuki , Y. Shimizu , and T. Shiina , “ATP‐Induced Contractile Response of Esophageal Smooth Muscle in Mice,” International Journal of Molecular Sciences 25, no. 4 (2024): 1985, 10.3390/ijms25041985. 38396664 PMC10888660

[18] Z. Feng , M. E. Hom , T. E. Bearrood , et al., “Targeting Colorectal Cancer with Small‐Molecule Inhibitors of ALDH1B1,” Nature Chemical Biology 18, no. 10 (2022): 1065–1075, 10.1038/s41589-022-01048-w. 35788181 PMC9529790

[19] I. Tsochantaridis , D. Brisimis , M. Tsifintaris , et al., “Exploring the Role and Pathophysiological Significance of Aldehyde Dehydrogenase 1B1 (ALDH1B1) in Human Lung Adenocarcinoma,” International Journal of Molecular Sciences 25, no. 19 (2024): 10301, 10.3390/ijms251910301. 39408636 PMC11477306

[20] X. Chen , J. Huang , C. Yu , et al., “A Noncanonical Function of EIF4E Limits ALDH1B1 Activity and Increases Susceptibility to Ferroptosis,” Nature Communications 13, no. 1 (2022): 6318, 10.1038/s41467-022-34096-w. PMC9588786 36274088

[21] B. C. Jackson , P. Reigan , B. Miller , D. C. Thompson , and V. Vasiliou , “Human ALDH1B1 Polymorphisms May Affect the Metabolism of Acetaldehyde and All‐Trans Retinaldehyde—In Vitro Studies and Computational Modeling,” Pharmaceutical Research 32, no. 5 (2015): 1648–1662, 10.1007/s11095-014-1564-3. 25413692 PMC4382438

[22] G. Bassotti , G. de Roberto , D. Castellani , L. Sediari , and A. Morelli , “Normal Aspects of Colorectal Motility and Abnormalities in Slow Transit Constipation,” World Journal of Gastroenterology 11, no. 18 (2005): 2691–2696, 10.3748/wjg.v11.i18.2691. 15884105 PMC4305899

[23] Y. Fan , C. Xu , L. Xie , et al., “Abnormal Bile Acid Metabolism Is an Important Feature of Gut Microbiota and Fecal Metabolites in Patients with Slow Transit Constipation,” Frontiers in Cellular and Infection Microbiology 12 (2022): 956528, 10.3389/fcimb.2022.956528. 35967856 PMC9366892

[24] N. J. Spencer and H. Hu , “Enteric Nervous System: Sensory Transduction, Neural Circuits and Gastrointestinal Motility,” Nature Reviews Gastroenterology & Hepatology 17, no. 6 (2020): 338–351, 10.1038/s41575-020-0271-2. 32152479 PMC7474470

[25] R. Hamnett , J. L. Bendrick , Z. Saha , et al., “Enteric Glutamatergic Interneurons Regulate Intestinal Motility,” Neuron 113, no. 7 (2025): 1019–1035, 10.1016/j.neuron.2025.01.014. 39983724 PMC11968238

[26] J. D. Windster , N. J. M. Kakiailatu , L. E. Kuil , et al., “Human Enteric Glia Diversity in Health and Disease: New Avenues for the Treatment of Hirschsprung Disease,” Gastroenterology 168, no. 5 (2025): 965–979, 10.1053/j.gastro.2024.12.011. 39725172

[27] N. Israelyan , A. Del Colle , Z. Li , et al., “Effects of Serotonin and Slow‐Release 5‐Hydroxytryptophan on Gastrointestinal Motility in a Mouse Model of Depression,” Gastroenterology 157, no. 2 (2019): 507–521, 10.1053/j.gastro.2019.04.022. 31071306 PMC6650329

[28] J. B. Furness , “The Enteric Nervous System and Neurogastroenterology,” Nature Reviews Gastroenterology & Hepatology 9, no. 5 (2012): 286–294, 10.1038/nrgastro.2012.32. 22392290

[29] A. Paul , C. Corbett , and A. Peluzzo , “FXR1 regulates Vascular Smooth Muscle Cell Cytoskeleton, VSMC Contractility, and Blood Pressure by Multiple Mechanisms,” Cell reports 42, no. 4 (2023): 112381, 10.1016/j.celrep.2023.112381. 37043351 PMC10564969

[30] T. H. Kwon , H. Jung , E. J. Cho , J. H. Jeong , and U. D. Sohn , “The Signaling Mechanism of Contraction Induced by ATP and UTP in Feline Esophageal Smooth Muscle Cells,” Molecules and Cells 38, no. 7 (2015): 616–623, 10.14348/molcells.2015.2357. 26013385 PMC4507027

[31] R. Tian , X. Li , J. Su , et al., “Regional Uterine Contractility Differences during Pregnancy: the Role of Hypoxia and Ferroptosis in Vitro,” Life Sciences 371 (2025): 123603, 10.1016/j.lfs.2025.123603. 40185467

[32] T. Ding , S. Wang , X. Zhang , et al., “Kidney Protection Effects of Dihydroquercetin on Diabetic Nephropathy through Suppressing ROS and NLRP3 Inflammasome,” Phytomedicine 41 (2018): 45–53, 10.1016/j.phymed.2018.01.026. 29519318

[33] H. Liu , Q. Hu , K. Ren , P. Wu , Y. Wang , and C. Lv , “ALDH2 mitigates LPS‐Induced Cardiac Dysfunction, Inflammation, and Apoptosis through the cGAS/STING Pathway,” Molecular Medicine 29, no. 1 (2023): 171, 10.1186/s10020-023-00769-5. 38124089 PMC10731778

[34] M. Hargreaves and L. L. Spriet , “Skeletal Muscle Energy Metabolism during Exercise,” Nature Metabolism 2, no. 9 (2020): 817–828, 10.1038/s42255-020-0251-4. 32747792

[35] G. Gherardi , A. Weiser , F. Bermont , et al., “Mitochondrial Calcium Uptake Declines during Aging and Is Directly Activated by Oleuropein to Boost Energy Metabolism and Skeletal Muscle Performance,” Cell Metabolism 37, no. 2 (2025): 477–495, 10.1016/j.cmet.2024.10.021. 39603237

[36] S. Zuo , B. Wang , J. Liu , et al., “ER‐Anchored CRTH2 Antagonizes Collagen Biosynthesis and Organ Fibrosis via Binding LARP6,” The EMBO Journal 40, no. 16 (2021): 107403, 10.15252/embj.2020107403. PMC8365266 34223653

[37] D. Peng , M. Fu , M. Wang , Y. Wei , and X. Wei , “Targeting TGF‐β Signal Transduction for Fibrosis and Cancer Therapy,” Molecular Cancer 21, no. 1 (2022): 104, 10.1186/s12943-022-01569-x. 35461253 PMC9033932

[38] M. Parola and M. Pinzani , “Liver Fibrosis in NAFLD/NASH: from Pathophysiology towards Diagnostic and Therapeutic Strategies,” Molecular Aspects of Medicine 95 (2024): 101231, 10.1016/j.mam.2023.101231. 38056058

[39] L. Li , H. Fu , and Y. Liu , “The Fibrogenic Niche in Kidney Fibrosis: Components and Mechanisms,” Nature Reviews Nephrology 18, no. 9 (2022): 545–557, 10.1038/s41581-022-00590-z. 35788561

[40] P. Dourlen , D. Kilinc , I. Landrieu , J. Chapuis , and J. C. Lambert , “BIN1 and Alzheimer's Disease: The Tau Connection,” Trends in Neurosciences 48, no. 5 (2025): 349–361, 10.1016/j.tins.2025.03.004. 40268578

[41] H. Perdreau‐Dahl , D. B. Lipsett , M. Frisk , et al., “BIN1, Myotubularin, and Dynamin‐2 Coordinate T‐Tubule Growth in Cardiomyocytes,” Circulation Research 132, no. 11 (2023): e188–e205, 10.1161/CIRCRESAHA.122.321732. 37139790

[42] X. X. Jiang , Y. R. Zhu , H. M. Liu , S. L. Chen , and D. M. Zhang , “Effect of BIN1 on Cardiac Dysfunction and Malignant Arrhythmias,” Acta Physiologica 228, no. 3 (2020): 13429, 10.1111/apha.13429. 31837094

[43] L. L. Smith , V. A. Gupta , and A. H. Beggs , “Bridging Integrator 1 (Bin1) Deficiency in Zebrafish Results in Centronuclear Myopathy,” Human Molecular Genetics 23, no. 13 (2014): 3566–3578, 10.1093/hmg/ddu067. 24549043 PMC4049309

[44] D. Stagos , Y. Chen , C. Brocker , et al., “Aldehyde Dehydrogenase 1B1: Molecular Cloning and Characterization of a Novel Mitochondrial Acetaldehyde‐Metabolizing Enzyme,” Drug Metabolism and Disposition 38, no. 10 (2010): 1679–1687, 10.1124/dmd.110.034678. 20616185 PMC2957164

[45] I. Tsochantaridis , A. Roupas , S. Mohlin , A. Pappa , and G. P. Voulgaridou , “The Concept of Cancer Stem Cells: Elaborating on ALDH1B1 as an Emerging Marker of Cancer Progression,” Life 13, no. 1 (2023): 197, 10.3390/life13010197. 36676146 PMC9863106

[46] E. Mameishvili , I. Serafimidis , S. Iwaszkiewicz , et al., “Aldh1b1 expression Defines Progenitor Cells in the Adult Pancreas and Is Required for Kras‐Induced Pancreatic Cancer,” Proceedings of the National Academy of Sciences 116, no. 41 (2019): 20679–20688, 10.1073/pnas.1901075116. PMC6789925 31548432

[47] R. Zhao , Z. Guo , K. Lu , et al., “Hepatocyte‐Specific NR5A2 Deficiency Induces Pyroptosis and Exacerbates Non‐Alcoholic Steatohepatitis by Downregulating ALDH1B1 Expression,” Cell Death & Disease 15, no. 10 (2024): 770, 10.1038/s41419-024-07151-1. 39438459 PMC11496806

[48] J. I. Lee , H. Park , M. A. Kamm , and I. C. Talbot , “Decreased Density of Interstitial Cells of Cajal and Neuronal Cells in Patients with Slow‐Transit Constipation and Acquired Megacolon,” Journal of Gastroenterology and Hepatology 20, no. 8 (2005): 1292–1298, 10.1111/j.1440-1746.2005.03809.x. 16048580

[49] Q. He , C. Han , L. Huang , et al., “Astragaloside IV Alleviates Mouse Slow Transit Constipation by Modulating Gut Microbiota Profile and Promoting Butyric Acid Generation,” Journal of Cellular and Molecular Medicine 24, no. 16 (2020): 9349–9361, 10.1111/jcmm.15586. 32628809 PMC7417726

[50] S. Givvimani , C. Munjal , N. Narayanan , et al., “Hyperhomocysteinemia Decreases Intestinal Motility Leading to Constipation,” American Journal of Physiology‐Gastrointestinal and Liver Physiology 303, no. 3 (2012): G281–G290, 10.1152/ajpgi.00423.2011. 22595990 PMC3423105

[51] M. Manetti , I. Rosa , L. Messerini , and L. Ibba‐Manneschi , “Telocytes Are Reduced during Fibrotic Remodelling of the Colonic Wall in Ulcerative Colitis,” Journal of Cellular and Molecular Medicine 19, no. 1 (2015): 62–73, 10.1111/jcmm.12457. 25283476 PMC4288350

[52] X. He , K. Dong , J. Shen , et al., “The Long Noncoding RNA Cardiac Mesoderm Enhancer‐Associated Noncoding RNA (Carmn) Is a Critical Regulator of Gastrointestinal Smooth Muscle Contractile Function and Motility,” Gastroenterology 165, no. 1 (2023): 71–87, 10.1053/j.gastro.2023.03.229. 37030336 PMC10330198

[53] Y. Tao , M. Liu , G. Siebert , et al., “I‐mfa, Mesangial Cell TRPC1 Channel, and Regulation of GFR,” Journal of the American Society of Nephrology 36, no. 4 (2025): 614–627, 10.1681/ASN.0000000533. 39446484 PMC11975231

[54] Y. Shi , Y. Li , J. Yin , et al., “A Novel Sympathetic Neuronal GABAergic Signalling System Regulates NE Release to Prevent Ventricular Arrhythmias after Acute Myocardial Infarction,” Acta Physiologica 227, no. 2 (2019): 13315, 10.1111/apha.13315. PMC6813916 31116911

[55] J. A. Murphy , D. N. Criddle , M. Sherwood , et al., “Direct Activation of Cytosolic Ca^2+^ Signaling and Enzyme Secretion by Cholecystokinin in human Pancreatic Acinar Cells,” Gastroenterology 135, no. 2 (2008): 632–641, 10.1053/j.gastro.2008.05.026. 18555802

[56] J. Liu , K. Liu , Y. Wang , et al., “Death Receptor 5 Is Required for Intestinal Stem Cell Activity during Intestinal Epithelial Renewal at Homoeostasis,” Cell Death & Disease 15, no. 1 (2024): 27, 10.1038/s41419-023-06409-4. 38199990 PMC10782029

[57] E. W. Deutsch , N. Bandeira , Y. Perez‐Riverol , et al., “The ProteomeXchange Consortium at 10 Years: 2023 Update,” Nucleic Acids Research 51, no. D1 (2023): D1539–D1548, 10.1093/nar/gkac1040. 36370099 PMC9825490

[58] Y. Perez‐Riverol , C. Bandla , D. J. Kundu , et al., “The PRIDE Database at 20 Years: 2025 Update,” Nucleic Acids Research 53, no. D1 (2025): D543–D553, 10.1093/nar/gkae1011. 39494541 PMC11701690

