# Supplementary material for: BIN1 and ALDH1B1 Deficiency in Colonic Smooth Muscle Drives Mitochondrial Dysfunction and Fibrosis in Slow‐Transit Constipation
Source: Adv Sci (Weinh). 2026 Jun 12;13(50):e23688. doi: 10.1002/advs.202523688 (PMC13336480; doi:10.1002/advs.202523688)
Supplement: Supplementary file 1 [file ADVS-13-e23688-s001.docx]

**Supporting Information**

**Title**

**BIN1 and ALDH1B1 deficiency in colonic smooth muscle drives mitochondrial dysfunction and fibrosis in slow-transit constipation**

***Author(s) ^#^, and Corresponding Author(s)*** ***

*Jianbo Liu^1, 3#^, Hao Zhang^1#^*, Wenhao Qiao^1^, Ran Liu^1^, Jie Sun^1^, Xiaopei Li^2^, Qin Li^3^, Dongbo Zhao^4^, Dawei Chen^5, 6, 7^*,* *Jingxin Li^3, 8^*, Shuxiao Dong^1^**

**Figure S1. Damage to the enteric nervous system in STC patients led to intestinal motility dysfunction**

Data are expressed as mean ± SEM. Statistical analyses were performed by the paired two-tailed t-test. Differences were considered statistically significant at *p* < 0.05*.*

**A**. Volcano plots visualized the DEPs between pathological region and normal region of ENS in colon tissues from STC patients. DEPs (*p* value < 0.05 and P_ENS/N_ENS Ratio > 1.5 or < 2/3) were highlighted in red (upregulated in P_ENS group) and blue (downregulated in P_ENS group).

**B**. Immunofluorescence images of TUBB3 and CADM2 in colon tissues with pathological region and normal region from STC patients (scale bar: 100 μm). Yellow arrows pointed CM, LM, and ENS structure regions of colon tissues.

**C**. Immunofluorescence images of TUBB3 and KCTD12 in colon tissues with pathological region and normal region from STC patients (scale bar: 100 μm). Yellow arrows pointed CM, LM, and ENS structure regions of colon tissues.

**D**. Statistical graphs showing the fluorescence intensity of CADM2 and KCTD12 between pathological region and normal region of ENS in colon tissues from STC patients (n=6).

**E**. Western blot showing the protein expression of CADM2 and KCTD12 between pathological region and normal region of ENS in colon tissues from STC patients (n=6).

**F**. Circle plot showing the selected GOBP terms for the enrichment of downregulated protein clusters in ENS. The enriched terms related to ENS structure and function were highlighted in red.

**G**. Bar graph showing the significantly enriched KEGG pathways for downregulated proteins in ENS. The enriched terms related to ENS structure and function were highlighted in red.

**Figure S2. Validation of spatial proteomics data in the CM** **of colon tissues**

Data are expressed as mean ± SEM. Statistical analyses were performed by the paired two-tailed t-test. Differences were considered statistically significant at *p* < 0.05*.*

**A.** Immunofluorescence images of SMMHC and FABP3 in colon tissues with pathological region and normal region from STC patients (scale bar: 100 μm). Yellow arrows pointed CM, LM, and ENS structure regions of colon tissues.

**B.** Statistical graphs showing the fluorescence intensity of FABP3 between pathological region and normal region of CM in colon tissues from STC patients (n=6).

**C.** Western blot showing the protein expression of FABP3 between pathological region and normal region of CM in colon tissues from STC patients (n=6).

**Figure S3. Bin1 knockdown enhances susceptibility to STC induced by loperamide in mice**

Data are expressed as mean ± SEM. Each experimental group includes data from at least six independent mice. Statistical analyses were performed by the unpaired two-tailed t-test. Differences were considered statistically significant at *p* < 0.05.

**A.** The experimental design.

**B.** qRT-PCR showing the mRNA expression of Bin1 in the colonic circular muscle of mice from Control, Loperamide and sh-Bin1 + Loperamide groups (n=6).

**C and D.** Western blot showing the protein expression of BIN1 in the colonic circular muscle of mice from Control, Loperamide and sh-Bin1 + Loperamide groups (n=6).

**E-H.** The effect of Bin1 knockdown on (E) fecal output, (F) pellet water content, (G) whole gut transit time, and (H) colonic transit time in SPF mice with STC induced by loperamide (n=10).

**I.** qRT-PCR showing the mRNA expression of the smooth muscle contraction related genes in the colonic circular muscle of mice from Control, Loperamide and sh-Bin1 + Loperamide groups (n=6).

**Figure S4. Restoring BIN1 expression rescues the contractile dysfunction in HCoSMCs induced by its knockdown**

Data are expressed as mean ± SEM. Data from in vitro assays are representative of at least three independent experiments. Statistical analyses were performed by the unpaired two-tailed t-test. Differences were considered statistically significant at *p* < 0.05*.*

**A.** qRT-PCR showing the mRNA expression of BIN1 in HCoSMCs from NC, BIN1-KD, and BIN1-KD + OE groups (n=6). KD, knockdown; OE, overexpression.

**B.** Western blot showing the protein expression of BIN1 in HCoSMCs from NC, BIN1-KD, and BIN1-KD + OE groups (n=6).

**C.** Pictures showing the change of cell surface area during the photography period. The representative pictures showed the 0‐ and 15‐minute results after treatment with ATP (10 µM).

**D.** Statistical graphs showing the change rate of cell surface area after treatment with ATP (n=3). Cell contraction was determined by the changes in the planar surface area. The percent decrease in surface area was calculated as [(the surface area of cell after ATP–the surface area of the cell before ATP)/the surface area before ATP] ×100% using ImageJ software.

**E.** qRT-PCR showing the mRNA expression of the smooth muscle contraction related genes in HCoSMCs from NC, BIN1-KD, and BIN1-KD + OE groups (n=6).

**Figure S5. Validation of spatial proteomics data in the LM** **of colon tissues**

Data are expressed as mean ± SEM. Statistical analyses were performed by the paired two-tailed t-test. Differences were considered statistically significant at *p* < 0.05*.*

**A**. Immunofluorescence images of SMMHC and SLMAP in colon tissues with pathological region and normal region from STC patients (scale bar: 100 μm). Yellow arrows pointed CM, LM, and ENS structure regions of colon tissues.

**B**. Statistical graphs showing the fluorescence intensity of SLMAP between pathological region and normal region of LM in colon tissues from STC patients (n=6).

**C**. Western blot showing the protein expression of SLMAP between pathological region and normal region of LM in colon tissues from STC patients (n=6).

**Figure S6. Aldh1b1 knockdown enhances susceptibility to STC induced by loperamide in mice**

Data are expressed as mean ± SEM. Each experimental group includes data from at least six independent mice. Statistical analyses were performed by the unpaired two-tailed t-test. Differences were considered statistically significant at *p* < 0.05.

**A.** The experimental design.

**B.** qRT-PCR showing the mRNA expression of Aldh1b1 in the colonic longitudinal muscle of mice from Control, Loperamide and sh-Aldh1b1 + Loperamide groups (n=6).

**C and D.** Western blot showing the protein expression of ALDH1B1 in the colonic longitudinal muscle of mice from Control, Loperamide and sh-Aldh1b1 + Loperamide groups (n=6).

**E-H.** The effect of Aldh1b1 knockdown on (E) fecal output, (F) pellet water content, (G) whole gut transit time, and (H) colonic transit time in SPF mice with STC induced by loperamide (n=9).

**I.** qRT-PCR showing the mRNA expression of the smooth muscle contraction related genes in the colonic longitudinal muscle of mice from Control, Loperamide and sh-Aldh1b1 + Loperamide groups (n=6).

**Figure S7. Restoring ALDH1B1 expression rescues the contractile dysfunction in HCoSMCs induced by its knockdown**

Data are expressed as mean ± SEM. Data from in vitro assays are representative of at least three independent experiments. Statistical analyses were performed by the unpaired two-tailed t-test. Differences were considered statistically significant at *p* < 0.05*.*

**A.** qRT-PCR showing the mRNA expression of ALDH1B1 in HCoSMCs from NC, ALDH1B1-KD, and ALDH1B1-KD + OE groups (n=6).

**B.** Western blot showing the protein expression of ALDH1B1 in HCoSMCs from NC, ALDH1B1-KD, and ALDH1B1-KD + OE groups (n=6).

**C.** Pictures showing the change of cell surface area during the photography period. The representative pictures showed the 0‐ and 15‐minute results after treatment with ATP (10 µM).

**D.** Statistical graphs showing the change rate of cell surface area after treatment with ATP (n=3). Cell contraction was determined by the changes in the planar surface area. The percent decrease in surface area was calculated as [(the surface area of cell after ATP–the surface area of the cell before ATP)/the surface area before ATP] ×100% using ImageJ software.

**E.** qRT-PCR showing the mRNA expression of the smooth muscle contraction related genes in HCoSMCs from NC, ALDH1B1-KD, and ALDH1B1-KD + OE group (n=6).

**Figure S8. BIN1 and ALDH1B1 both suppress the fibrotic progression of HCoSMCs by maintaining mitochondrial ROS homeostasis**

Data are expressed as mean ± SEM. All experiments were conducted with six independent biological replicates. Statistical analyses were performed by the unpaired two-tailed t-test. Differences were considered statistically significant at *p* < 0.05*.*

**A.** qRT-PCR showing the mRNA expression of the profibrotic response related genes in HCoSMCs from NC, BIN1-KD, and BIN1-KD + OE groups (n=6).

**B.** qRT-PCR showing the mRNA expression of the profibrotic response related genes in HCoSMCs from NC, sh-BIN1 and sh-BIN1 + MitoQ groups (n=6).

**C.** qRT-PCR showing the mRNA expression of the profibrotic response related genes in HCoSMCs from NC, ALDH1B1-KD, and ALDH1B1-KD + OE groups (n=6).

**D.** qRT-PCR showing the mRNA expression of the profibrotic response related genes in HCoSMCs from NC, sh-ALDH1B1 and sh-ALDH1B1 + MitoQ groups (n=6).

**Figure S9. The loss of Bin1 and Aldh1b1 both promote fibrosis of colonic smooth muscle cells in STC mice**

Data are expressed as mean ± SEM. All experiments were conducted with six independent biological replicates. Statistical analyses were performed by the unpaired two-tailed t-test. Differences were considered statistically significant at *p* < 0.05*.*

**A.** qRT-PCR showing the mRNA expression of the profibrotic response related genes in the colonic circular muscle of mice from Control, Loperamide and sh-Bin1 + Loperamide groups (n=6).

**B.** qRT-PCR showing the mRNA expression of the profibrotic response related genes in the colonic longitudinal muscle of mice from Control, Loperamide and sh-Aldh1b1 + Loperamide groups (n=6).

**C and D.** Representative images of Sirius Red staining (scale bar: 100 μm).

**E and F.** Representative images of Masson staining (scale bar: 100 μm).

**Table S1 The information of STC patients used in this study**

| **Number** | **Sex** | **Age** | **Height (cm)** | **Weight (kg)** | **Medical Record Number (MRN)** | **History of Constipation** | **Past Medical History** |
| --- | --- | --- | --- | --- | --- | --- | --- |
| **1** | Female | 54 | 163 | 70 | 323706 | over 20 years；  Long-term pharmacotherapy； | History of hypertension |
| **2** | Male | 83 | 175 | 75 | 326471 | over 30 years；  Long-term pharmacotherapy；  Worsening for half a month with intestinal obstruction | History of cerebral arteriosclerosis |
| **3** | Female | 64 | 155 | 50 | 362526 | over 10 years；  Long-term pharmacotherapy；  Worsening for half a year | History of rectal cancer surgery |
| **4** | Female | 47 | 160 | 58 | 326952 | over 5 years；  Long-term pharmacotherapy；  Worsening for a year | None |
| **5** | Male | 15 | 178 | 60 | 332949 | over 2 years；  Repeated episodes of intestinal obstruction for over six months | None |
| **6** | Male | 34 | 173 | 60 | 336973 | over 2 years；  Long-term pharmacotherapy；  Worsening for 3 months | History of hepatitis B |
| **7** | Female | 79 | 168 | 85 | 334874 | over 2 years；  Long-term pharmacotherapy；  Worsening with abdominal pain for one month | None |
| **8** | Female | 52 | 168 | 80 | 331293 | over 2 years；  Long-term pharmacotherapy;  Worsening with intestinal obstruction for 1 week | History of cesarean section |
| **9** | Female | 36 | 162 | 53 | 336456 | over 10 years；  Long-term pharmacotherapy；  Worsening for 2 years | History of left fallopian tube cyst surgery |
| **10** | Female | 76 | 166 | 70 | 336138 | over 5 years；  Long-term pharmacotherapy；  Worsening for 1 year | History of lumbar vertebral fracture surgery |
| **11** | Female | 77 | 155 | 50 | 336261 | over 30 years；  Long-term pharmacotherapy；  Worsening with intestinal obstruction for 2 weeks | History of diabetes |
| **12** | Male | 59 | 175 | 58.5 | 334659 | over 7 years；  Long-term pharmacotherapy；  Worsening for 1 year | None |
| **13** | Male | 61 | 166 | 35 | 336656 | over 2 years；  Long-term pharmacotherapy； | History of rectal cancer surgery |
| **14** | Male | 86 | 160 | 45 | 337336 | over 20 years；  Long-term pharmacotherapy；  Worsening for 1 year | History of intestinal obstruction surgery |
| **15** | Female | 50 | 150 | 52 | 354920 | over 8 years；  Long-term pharmacotherapy； | History of diabetes |
| **16** | Male | 67 | 160 | 70 | 354426 | over 20 years；  Long-term pharmacotherapy； | History of thyroid surgery |
| **17** | Female | 47 | 157 | 51 | 355728 | Half a year；Worsening for half a month | None |
| **18** | Female | 61 | 161 | 71 | 358875 | over 7 years；  Long-term pharmacotherapy；  Worsening for half a year | None |
| **19** | Male | 58 | 165 | 71 | 359875 | One month；  Recurrent intestinal obstruction | History of hypertension;  History of diabetes |
| **20** | Female | 74 | 150 | 40 | 361118 | over 2 years；  Long-term pharmacotherapy； | History of hypertension;  History of diabetes |
| **21** | Female | 53 | 165 | 55.5 | 361238 | over 30 years；Long-term pharmacotherapy； | None |
| **22** | Female | 33 | 160 | 55 | 341667 | over 10 years；  Long-term pharmacotherapy； | None |
| **23** | Female | 71 | 168 | 49 | 342914 | over 20 years；  Long-term pharmacotherapy； | None |
| **24** | Female | 40 | 172 | 50 | 345742 | over 10 years；Long-term pharmacotherapy； | History of right breast nodule surgery |
| **25** | Female | 49 | 160 | 58.5 | 346109 | over 40 years；Long-term pharmacotherapy； | History of cesarean delivery |
| **26** | Female | 70 | 160 | 52 | 347797 | over 30 years；Long-term pharmacotherapy； | History of diabetes |
| **27** | Female | 34 | 165 | 65 | 349707 | over 20 years；  Long-term pharmacotherapy； | None |
| **28** | Female | 60 | 163 | 56 | 349894 | over 20 years；Long-term pharmacotherapy； | None |
| **29** | Female | 52 | 155 | 48 | 350858 | over 2 years；  Long-term pharmacotherapy | History of cesarean section surgery |
| **30** | Male | 49 | 170 | 60 | 348322 | over 3 years；  Long-term pharmacotherapy； | History of diabetes |
| **31** | Male | 69 | 170 | 60 | 340263 | over 20 years；  Long-term pharmacotherapy； | History of hypertension; History of cerebral infarction |
| **32** | Female | 45 | 165 | 52.5 | 362332 | over 40 years；  Long-term pharmacotherapy； | None |
| **33** | Female | 42 | 160 | 56 | 356998 | over 10 years；  Long-term pharmacotherapy； | None |
| **34** | Female | 38 | 155 | 55 | 355860 | over 5 years；  Long-term pharmacotherapy； | None |
| **35** | Female | 41 | 162 | 60 | 361153 | over 3 years；  Long-term pharmacotherapy； | History of cesarean section surgery;  History of salpingectomy |

**Table S2 Primer genes used in running qRT-PCR along with their forward and reverse sequences**

| Primers | Forward Sequence | Reverse Sequence |  |
| --- | --- | --- | --- |
| CYR61 | CCAGTGTACAGCAGCCTGAA | CGCATCTTCACAGTCCTGGT | |
| MYH9 | CCUCGAGAAGGCAAGGCAGA | CACTTTCTTGCTGCTTGTGCT |  |
| IL6 | AGTGAGGAACAAGCCAGAGC | GGTCAGGGGTGGTTATTGCA |  |
| SERPINE1 | GCAACGTGGTTTTCTCACCC | ATCTGCTGCTGGGTTTCTCC |  |
| THBS1 | TTTGGCTACCAGTCCAGCAG | AGAAAGGCCCGAGTATCCCT |  |
| TGFB2 | GCGCTACATCGACAGCAAAG | TGCAGCAGGGACAGTGTAGG |  |
| CTGF | TGGAGTTCAGGTGCCCTGAC | TCCGGGACAGTTGTAATGGG |  |
| FN1 | AGCCTGGGAGCTCTATTCCA | CTTGGTCGTACACCCAGCTT |  |
| COL1A2 | CTCCTGGGCTGAGAGGTAGT | ACTTGCACCACGACTACCAG |  |
| FAP | GTATGGTGGTCCCTGCAGTC | CCAAGGCAATGACCATCCCT |  |
| CCL5 | CAAGGGCAAGGTTTGTGACC | GCCTCCCAAGCTAGGACAAG |  |
| SOX9 | TTCACCTACATGAACGCCGC | AGCTGTGTGTAGACGGGTTG |  |
| TGFBR2 | CAACCACAACACAGAGCTGC | GTGTTCTGCTTCAGCTTGGG |  |
| ECM1 | GAGTCACCCCCAACCTCATG | ATGTTGTGGATCAGCCCAGG |  |
| SERPINE2 | AAGAAACGCACTTTCGTGGC | CCGTGGTAGGGCAGTTCAAT |  |
| TGFBI | CGAGGACACCTTTGAGACCC | AGGGATCTTCTCGAAGGCCT |  |
| COL6A1 | CACTCAGAGGGACACCACAC | ACATTGAGCTGGTCTGAGCC |  |
| SLC25A41 | AGGGCTCAAAATCCCACCATG | ATGCCACCTGCTGGTAAGAC |  |
| SLC25A32 | TGTCGCAGCAACATACCCAT | AATCCACCGACGCCTTCTTT |  |
| ATP6V1G2 | ATGCCAGAAAGAGGAAGGCC | ACACGCTCTCGGTTTCTCTG |  |
| TRPM6 | GTCTTGCCTCTGTCCAGACC | GGTTCATCCACTGATGCCCA |  |
| TRPM3 | TCGGTCAAGGTGTTCCTGTG | AAAGGCCAGGATGTCCGATG |  |
| CLCA2 | GGACAGCACCTGGAGAAGAC | ATGCCAGCTTGCTGAGGATT |  |
| CYCS | CGTTGAAAAGGGAGGCAAGC | ATTGGCGGCTGTGTAAGAGT |  |
| UQCRC1 | AGGTTAGCCTGCTGGACAAC | CAAAACGGCTGCCAACATCA |  |
| SDHB | TGGGGCCTGCAGTTCTTATG | GAGAATGGGTCCTGCAGCTT |  |
| NDUFS3 | TGGAGTTGGCCCAAGAGTTC | CTCCGGCTTCAAGCTTGAGA |  |
| TFAM | GAAGAATTGCCCAGCGTTGG | CTGCCACTCCGCCCTATAAG |  |
| CAV1 | GCAGAACCAGAAGGGACACACAG | CCAAAGAGGGCAGACAGCAAGC |  |
| SYNM | GCAGAAGTGGTGGAGATGGATGTG | ACTGCCTGGTCATTCCTACTGTCC |  |
| TAGLN | TCTGGCTGAAGAATGGCGTGATTC | CCACCTGCTCCATCTGCTTGAAG |  |
| ALDH1B1 | CCTGGCTGCGGCTGTGTTC | GTGGCAGGTGACGATGTTGTAGG |  |
| BIN1 | TCAGAGTCAACCACGAGCCAGAG | TCCACCACGACAGCAGGAAGAG |  |
| TPM1 | CGACAAGAAGGCGGCGGAAG | CCAGTTCATCTTCGGTGCCCTTG |  |
| CHRM2 | AGCCCGCAAGATTGTGAAGATGAC | GCCCAAGTGATGATGAAAGCCAAC |  |
| CHRM3 | ACAGCAGCAGTGACAGTTGGAAC | GCTTGAGCACGATGGAGTAGATGG |  |
| GAPDH | GTGGACCTGACCTGCCGTCTAG | GAGTGGGTGTCGCTGTTGAAGTC |  |
| ACTG2 | GCAGGCTTCGCAGGAGATGATG | CGCAGCTCATTGTAGAAGGAGTGG |  |
| Tpm1 | GCAAATGTGCCGAGCTTGAA | CAGCCTCCTTCAGCTTGTCA |  |
| Synm | CTCCTGCAGGTGAAGACTGG | CTCTCGTGTCGCTCAGATCC |  |
| Cav1 | GACCCCAAGCATCTCAACGA | AGATGCCGTCGAAACTGTGT |  |
| Bin1 | CCTACCTAGGCCAGTTCCCT | CTCGAACACCTTCTGGGCTT |  |
| Gapdh | TGATGGGTGTGAACCACGAG | AGTGATGGCATGGACTGTGG |  |
| Thbs1 | CGCCTTCCGCATTGAGAATG | CATCTGCCTCAAGGAAGCCA |  |
| Tgfb2 | CAAGAGGATCGAGGCCATCC | GCTTGCCTTCTCCTGCAGTA |  |
| Serpine1 | TGAGAAGGGCACAGCTCATG | AGAGCTTGAAGAAGTGGGGC |  |
| Myh9 | CAGCAGCTGTTCAACCACAC | CATCTAGCAGGGCCAGGATG |  |
| Fn1 | CGTGATCATCGATGCCTCCA | AGGGGATCCAGGCTTCTCAT |  |
| Cyr61 | AGAGGCTTCCTGTCTTTGGC | CCAAGACGTGGTCTGAACGA |  |
| Ctgf | AGAACTGTGTACGGAGCGTG | CTTTGGAAGGACTCACCGCT |  |
| Chrm3 | ACTATGTGGCCAGCAATGCT | CGGCTCGTTTTGTTGTTCGT |  |
| Chrm2 | CTGGCCTTTGGGACCTGTAG | CTGCAATCATCATGCCTGCC |  |
| Tgfbr2 | GAGACTGTCCACTTGCGACA | TGTCGTTCTTCCTCCACACG |  |
| Tgfbi | ATGGAGATCCTCAACCGGGA | GTTCTTCTGGAGGCATGGCT |  |
| Sox9 | TCACTACAGCCCCTCCTACC | AGGTGGAGTAGAGCCCTGAG |  |
| Serpine2 | TGGCAGGTGATGGGAAATCC | GCTGATGCTCTCACCATGGT |  |
| Fap | CCTCCGAGCAAGAAGTGTGT | GCCATCATGGAGGGTGGAAA |  |
| Ecm1 | GGGAGGATGCAATGACCCAA | AGGACATGCCATTCTGGTGG |  |
| Col6a1 | GTGTGCTTGTCACTGGCAAG | GGTAGAGTACGCCACGTAGC |  |
| Col1a2 | ATTCCTGGTGCTGTAGGTGC | GCCAACTTCACCACGTTCAC |  |
| Tagln | GGTCCATCCTACGGCATGAG | CCTACATCAGGGCCACACTG |  |
| Ccl5 | TGAAGATCTCTGCAGCTGCC | GTCCGAGCCATATGGTGAGG |  |
| Actg2 | CCCAAAGCAAACAGAGAGAAGATGAC | GAGCACAGCCTGAATAGCAACATAC |  |
| Il6 | TCCTTCCTACCCCAATTTCCA | GTCTTGGTCCTTAGCCACTCC |  |
| Aldh1b1 | AGGGCAGGACTACCTCTTACT | CATGCCACTCGTTGTTGATGA |  |
